# Nonparametric Subgroup Identification by PRIM and CART: A Simulation and Application Study

**DOI:** 10.1155/2017/5271091

**Published:** 2017-05-22

**Authors:** Armin Ott, Alexander Hapfelmeier

**Affiliations:** Institute of Medical Statistics and Epidemiology, Technische Universität München, Ismaninger Str. 22, 81675 Munich, Germany

## Abstract

Two nonparametric methods for the identification of subgroups with outstanding outcome values are described and compared to each other in a simulation study and an application to clinical data. The Patient Rule Induction Method (PRIM) searches for box-shaped areas in the given data which exceed a minimal size and average outcome. This is achieved via a combination of iterative peeling and pasting steps, where small fractions of the data are removed or added to the current box. As an alternative, Classification and Regression Trees (CART) prediction models perform sequential binary splits of the data to produce subsets which can be interpreted as subgroups of heterogeneous outcome. PRIM and CART were compared in a simulation study to investigate their strengths and weaknesses under various data settings, taking different performance measures into account. PRIM was shown to be superior in rather complex settings such as those with few observations, a smaller signal-to-noise ratio, and more than one subgroup. CART showed the best performance in simpler situations. A practical application of the two methods was illustrated using a clinical data set. For this application, both methods produced similar results but the higher amount of user involvement of PRIM became apparent. PRIM can be flexibly tuned by the user, whereas CART, although simpler to implement, is rather static.

## 1. Introduction

Subgroup identification, especially in high-dimensional data situations, is a common problem. The aim is to find subsets of the whole data set defined by covariates in which the outcome of interest is distributed differently than in other regions. Especially in the medical domain, there are many possibilities for applications of methods that address this problem. For example, in the context of personalized medicine, subgroup identification can be of interest if a treatment effect is enhanced or reduced for groups of patients defined by the baseline covariates (cf. [1, 2]) or it may be desirable to find subgroups of patients with a high risk of mortality (cf. [3]). In addition to applications in medicine, there are also other fields in which such methods are useful such as industrial process control (cf. [4]).

The Patient Rule Induction Method (PRIM) and Classification and Regression Trees (CART) are two popular nonparametric methods for subgroup identification. They employ two different strategies which are described in this paper. PRIM, which is less commonly used, is explained in more detail in this paper. It formulates the research question as an optimization problem where some target function has to be maximized or minimized. A simple solution to this is to find specific values or regions for a set of variables (covariates) conditioned on which another variable (outcome) takes extreme values. This way, one tries to identify subgroups in the whole data set in which the mean outcome (or another criterion) is high or low. By contrast, CART provides an empirical description of the conditional distribution of an outcome as it splits the data into disjoint subsets. Some of these subsets may depict subgroups of interest to a focused research question. To assess the performance of PRIM and CART in subgroup identification, they were compared in different data settings in a simulation study and an application to clinical data. Corresponding R-codes are given in the supplementary Appendices C–G in Supplementary Material available online at https://doi.org/10.1155/2017/5271091.

## 2. The Patient Rule Induction Method (PRIM)

A PRIM model consists of boxes that define subsets (subgroups) with extreme outcome values. Boxes are defined by lower and upper threshold values for continuous covariates and subsets of the levels of categorical covariates. They are mainly characterized by their “target” and “support,” with the former being the result of the target function evaluated within the box and the latter describing the proportion of observations lying inside the box. Later in this section it will be shown that there is always a trade-off between those two values. A combination of two algorithms called “peeling” and “pasting” is used to fit the model in an iterative way (cf. [5, 6]).

### 2.1. Peeling

The main component of PRIM is the so-called top-down peeling. This iterative algorithm starts with a large box that contains all observations of a data set. Within every peeling step, small fractions (subboxes) are removed (peeled) from the margins of the current box, one at a time. Out of all these possible subboxes, the one which maximizes the target function on the remaining observations in the box is chosen for removal. If the goal is to minimize the target function, the algorithm acts the same way after multiplying the outcome *y* with the value −1 at the beginning so that the minimization problem is transformed into a maximization problem.

For most applications, the arithmetic mean is a useful choice for the target function:

(1) $$\begin{array}{c} f\left(\;y\;\right)=\frac{1}{n_{m+1}}\overset{n_{m+1}}{\underset{i=1}{\sum }}y_{i}. \end{array}$$

Here, *n*_*m*+1_ is the number of observations in the box:

(2) $$\begin{array}{c} B_{m+1}=B_{m}\setminus b_{m}^{*}, \end{array}$$

which results from the *m*th iterative step after a subbox *b*_*m*_^*∗*^ is chosen for removal out of the class of all possible subboxes *C*(*b*_*m*_) such that

(3) $$\begin{array}{c} b_{m}^{*}=\arg\underset{b_{m}\in \;C\left(b_{m}\right)}{max}f\left(\;y_{i}\;|\;x_{i}\in B_{m}\setminus b_{m}\;\right). \end{array}$$

In cases with only continuous covariates *x*_1_,…, *x*_*p*_, the set of possible subboxes *C*(*b*_*m*_) is composed as follows:

(4) $$\begin{array}{c} C\left(\;b_{m}\;\right)=\left\{\;b_{m1-},b_{m1+},b_{m2-},b_{m2+},\ldots ,b_{mp-},b_{mp+}\;\right\}, \end{array}$$

with

(5) bmj−=x ∣ xj≤xjmα,bmj+=x ∣ xj≥xjm1−α,

where *x*_*jm*(*α*)_ describes the *α*-quantile of the observations of variable *x*_*j*_ which lie in the current box *B*_*m*_.

Therefore, observations below the *α*-quantile or above the (1 − *α*)-quantile are peeled off and *α* can be seen as a metaparameter which is able to influence the result. Usually one chooses small values (0.05–0.1) which introduce the “patience” to the algorithm. *α* should be small enough that a potential suboptimal step does not have too much impact on the result but also not too small, because otherwise the boxes would depend strongly on the random variability in the data.

The peeling procedure is repeated until the support *β*_*m*_ of the current box *B*_*m*_ falls below some threshold *β*_0_, such that

(6) $$\begin{array}{c} \beta_{m}=\frac{1}{n}\overset{n}{\underset{i=1}{\sum }}I\left(\;x_{i}\in B_{m}\;\right)\leq \beta_{0}, \end{array}$$

where **I**(·) denotes the indicator function which returns the value 1, if the condition in brackets is true and 0 otherwise.

The minimum support *β*_0_ is another metaparameter which has to be determined by the user. The choice of this parameter depends on the analytic aims, but it should not be chosen too small, because very small boxes have strong dependency on the random noise in the data. Such a result would be very sensitive to small changes in the data set and prone to overfitting.

**Example 1 .**

**Figure 1:**
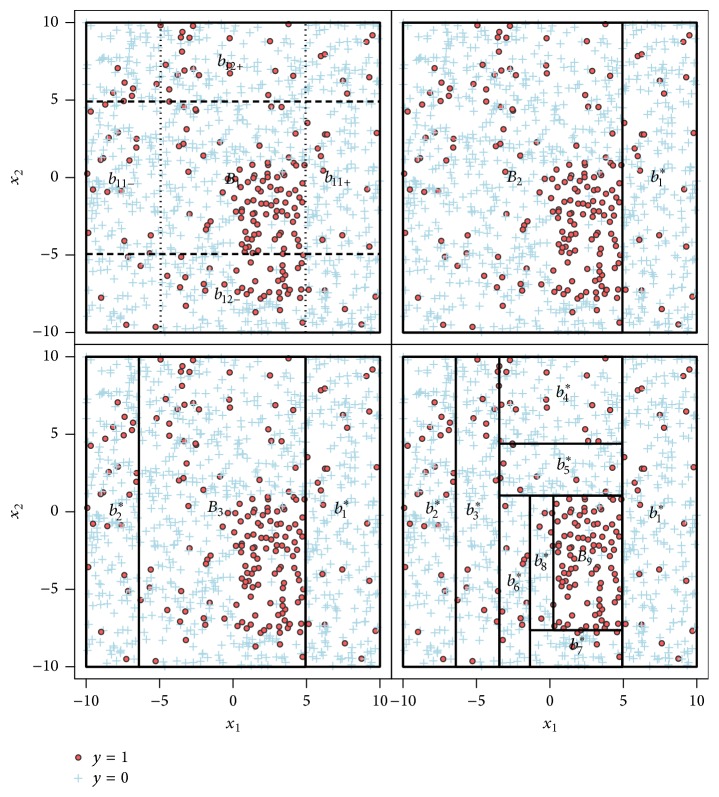
Example of a box sequence produced by the peeling algorithm with *α* = 0.25 and *β*_0_ = 0.075 for two covariates *X*_1_ and *X*_2_ and a binary outcome *Y*.

A simple example of the peeling algorithm and the sequence of boxes resulting out of it is illustrated in Figure 1. Here we have a binary outcome *Y* and two metric covariates *X*_1_ and *X*_2_ which are sampled from uniform distributions between −10 and 10. There is one obvious box in which the outcome is more frequent; therefore the mean outcome (0/1 coded) is much higher than for the rest of the data. To improve the appearance, *α* is chosen very high in this example at 0.25.

In the left upper panel, only the initial box *B*_1_ containing all data points and the four candidate boxes for the first peeling step are shown. The second and third graphs illustrate the first two steps of the algorithm with the two subboxes *b*_1_^*∗*^ and *b*_2_^*∗*^ peeled of the current box. The fourth one shows the result of the algorithm which is continued until *β*_0_ of 7.5% is reached, so *B*_9_ contains at least 7.5% of all observations. It is also clear to see that the subboxes become smaller with each step, because the *α*- and (1 − *α*)-quantiles refer only to the data that are included in the current box. In this case, the final box *B*_9_ is determined as

(7) $$\begin{array}{c} B_{9}=\left\{\begin{array}{cc} 0.23<x_{1}<4.94, & \\ -7.64<x_{2}<1.05. & \end{array}\right. \end{array}$$

#### 2.1.1. The Trajectory

A graphical illustration of the peeling steps is given by the so-called trajectory. It plots the value the target function takes at each iterative step against the corresponding box support. Users can judge a box to be “optimal” from this trade-off between mean outcome and box support.

**Figure 2:**
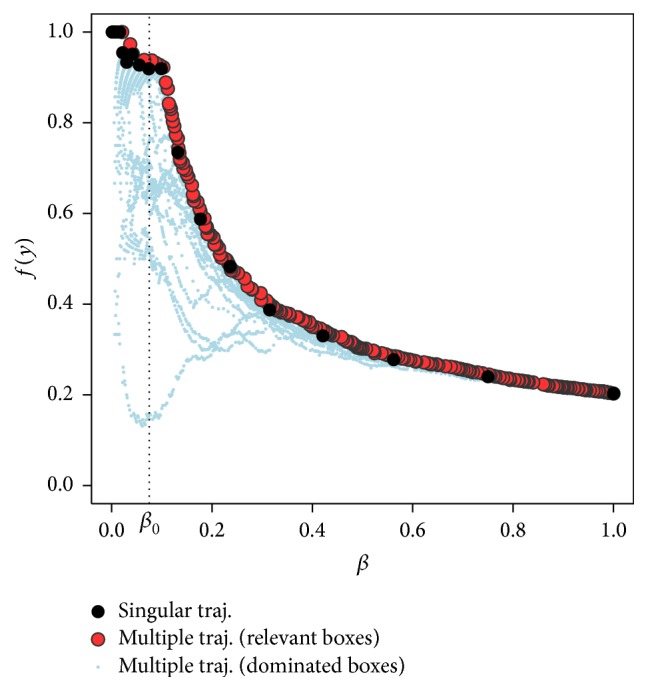
Singular trajectory for *α* = 0.25 and multiple trajectory for **α** = (0.01,0.05,0.1,0.2) and *s* = 10 bootstraps per *α*-fraction for sampled data.

The trajectory for the underlying example of Figure 1 is plotted in Figure 2 (black dots). What can be observed here is that the peeling starts with a box having a support of 1 and a box mean of about 0.2. As it continues, the support decreases and the target in most of the cases increases. In the current example, the minimum support *β*_0_ was carefully chosen at a point beyond which the box means do not get much larger any more so that it would not be advisable to continue peeling from there. Of course, in practice, it is not that simple, but the trajectory can still help the user to choose a box with properties that conform to specific requirements.

#### 2.1.2. Multiple Peeling

The trajectory can be unstable since it depends on metaparameters such as *α* and on random noise in the data. Different *α* values can lead to different trajectories, suggesting subboxes which may dominate each other in terms of support and mean outcome. A box *B*_*n*_ is said to be dominated by another box *B*_*m*_ if

(8) $$\begin{array}{c} f\left(\;y_{i}\;|\;x_{i}\in B_{n}\;\right)\leq f\left(\;y_{i}\;|\;x_{i}\in B_{m}\;\right),\;\beta_{n}<\beta_{m} \end{array}$$

or

(9) $$\begin{array}{c} f\left(\;y_{i}\;|\;x_{i}\in B_{n}\;\right)<f\left(\;y_{i}\;|\;x_{i}\in B_{m}\;\right),\;\beta_{n}\leq \beta_{m}. \end{array}$$

There are two options to perform multiple peeling. First, varying *α* values can be used, and, second, PRIM can be applied to bootstrapped samples of the data, which is called “bumping” (cf. [7, 8]). Generally, the best results can be achieved with a combination of both options. In this strategy, there are two metaparameters *s* and **α**, with the former being the number of bootstrap samples and the latter being a vector that describes a sequence of different peeling fractions. The parameters have to be determined by the user who now has to deal with a trade-off between computational effort and goodness of the result.

**Example 2 .**

An example for multiple trajectories is added in Figure 2 (coloured dots). The same data was used as in Figure 1. The metaparameters were set to **α** = (0.01,0.05,0.1,0.2) and *s* = 10, so, for the different *α*-fractions, peeling was applied once on the original data and 10 times on different bootstrap samples from it. After removing all dominated boxes that would not be chosen as a final box anyway, one gets a lucid figure (red dots) with only the relevant boxes. Again, the trajectory has an obvious peak at about *β*_0_ = 0.075. Dominated boxes of the multiple trajectory are illustrated by small blue dots.

### 2.2. Pasting

The so called bottom-up pasting is principally the complement of the peeling strategy. Starting with a box determined by peeling, this algorithm sequentially enlarges the box beyond its boundaries again. This way, the support increases and the target function could possibly increase too. Both are rated to be beneficial as PRIM is meant to find subgroups of sufficient size with increased average outcome. Such improvements by pasting are possible, because, during the peeling steps, decisions on boundaries are only locally optimal and conditional on the previous peeling steps. The algorithm does not look ahead on subsequent peeling steps. Therefore, the additive pasting procedure tries to correct on this shortcoming in order to approach a solution that is more globally optimal.

In pasting, the candidate subboxes to join the current one are defined equivalently to peeling. Another metaparameter *α*_paste_ defines the proportion of observations the subboxes contain. This value can differ from the *α* value that is used for the peeling. The box that maximizes the target function is finally chosen. Pasting continues until the target function on the data in the box decreases again (cf. [5]). Alternatively, pasting can be continued some steps after a possible decline to overcome local minima.

### 2.3. Covering

If one seeks to identify several subgroups, a strategy called “covering” is used. Observations included in a box are removed from the data set to make PRIM search for another one in the remaining parts. The procedure continues until some stop criterion is reached; for example, both values or either value of the target function and the support of boxes does not exceed some threshold. In addition to these criteria, it is also possible to define a maximum number of boxes. This is useful in cases when the user knows how many subgroups he wants to search for.

The final output is a set of boxes {*B*^(1)^,…, *B*^(*K*)^} which can be pooled to a larger region *R* = ⋃_*j*=1_^*K*^*B*^(*j*)^, if that is useful for the given situation. If the sequence of boxes is used for prediction, it can be seen as a “decision list” (cf. [9]). In this case, the prediction for a new observation would always be the box mean of the first box in the list it belongs to.

**Example 3 .**

**Figure 3:**
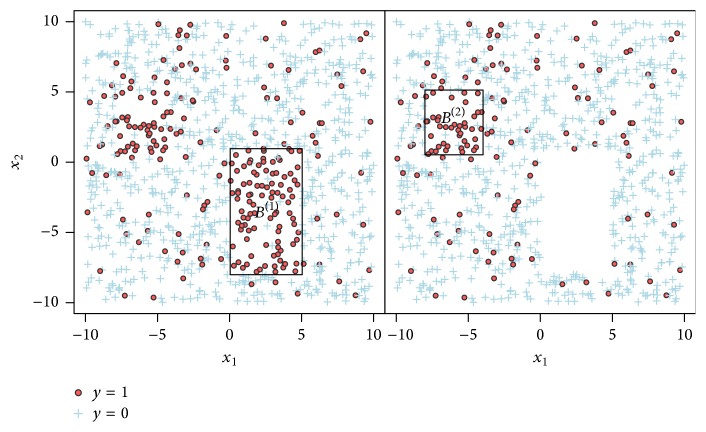
Illustration of the covering strategy for a binary outcome *Y* and two covariates *X*_1_ and *X*_2_.

A simple illustration of covering is pictured in Figure 3. The data here are similar to those in Figure 1 with the difference that now there are obviously two regions with an increased mean outcome $\bar{y}$. In this case, boxes with a minimum target of 0.9 having at least support of 0.01 were sought: *α* was set to 5% and *α*_paste_ was set to 1%.

### 2.4. User Involvement

An important factor that must not be underestimated in the application of PRIM is the user involvement. There are many possibilities to influence the method and, therefore, the final result. One of them is the definition of the metaparameters *α* and *β*_0_ (and *s*). Another is the decision on a box which is made by the user by looking at the (multiple) trajectory. The latter may be guided by prior knowledge about the size or the target of the sought subset. Furthermore, the user can decide on pasting steps, for instance, with the choice of *α*_paste_. The number of boxes to be found in the data is also determined by the user.

Any *α* and *α*_paste_ values can lead to a result that best suits an applicant's requirements. In that sense, they cannot serve as tuning parameters that could be optimized to find a “best” solution. Accordingly, it has been suggested in [5] to apply sets of alpha values and to use cross-validation to avoid overfitting issues.

An advantage of the strong user involvement is that it supports deliberate decision-making and leads to results that meet the users' needs. In addition, a user needs to make himself familiar with the given data situation and the interim results of the algorithm which may provide further information. An apparent disadvantage is that there needs to be sensible prior knowledge. Too much user involvement may also increase the risk of overfitting the algorithm to the given data.

## 3. Classification and Regression Trees (CART)

CART pursues goals similar to those of PRIM; that is, it also defines subsets in the data but uses a different strategy to do so. CART is a machine learning approach which fits prediction models to given data as it recursively splits the data into two disjoint parts by minimizing the heterogeneity of the outcome within each part. This heterogeneity is quantified by some impurity measure. The basic steps of the algorithm can be described as a short pseudocode as done in [10]:

1. Start at the root node (whole data set).
2. For all covariates *X*_*j*_, find the split *S* that minimizes the sum of the impurities in the two child nodes and choose that split *S*^*∗*^ which gives the minimum over all *X*_*j*_ and *S*.
3. Stop, if a given stopping criterion is reached; otherwise, run step 2 for each child node.

Classification trees are used for nominally scaled outcomes *y* that take *k* different values. Here, the impurity measure is the Gini index. Regression trees are fit to quantitative outcomes *y*. The impurity is measured by the residual sum of squares in that case.

The resulting model can be illustrated by a decision tree. A corresponding example is given for the application study in Figure 8. The output is similar to PRIM, since it defines subsets, which explains the trees' popularity for subgroup identification. CART is implemented by the function rpart() in the R-package rpart (cf. [11]).

## 4. Comparison of PRIM and CART

### 4.1. Simulation Study

#### 4.1.1. Study Design

The following simulation study was performed to compare PRIM with the alternative method CART with respect to their performance in identifying subgroups. In this section, the basic structure of such studies is described and possible factors that are able to influence the results are mentioned. Some factors that can potentially be modified between the simulation runs are the number of observations (*n*), the number of covariates (*p*), the scaling of covariates, the covariance of covariates (covariance matrix Σ), the scale of the outcome, the number of existent subgroups, the complexity of subgroups, the position of subgroups, and the signal-to-noise ratio (effect size versus random variability).

For this study, different numbers of simulated observations (*n* = {250,500,1000}) were sampled and for each of these observations six quantitative input variables *X*_1_,…, *X*_6_ were generated from uniform distributions:

(10) $$\begin{array}{c} X_{j}\overset{i.i.d.}{\sim }U\left(\;-1,1\;\right)\;j=1,\ldots ,6. \end{array}$$

In this scenario, *X*_1_,…, *X*_6_ are independent from each other, which means that no covariance structure is assumed.

**Figure 4:**
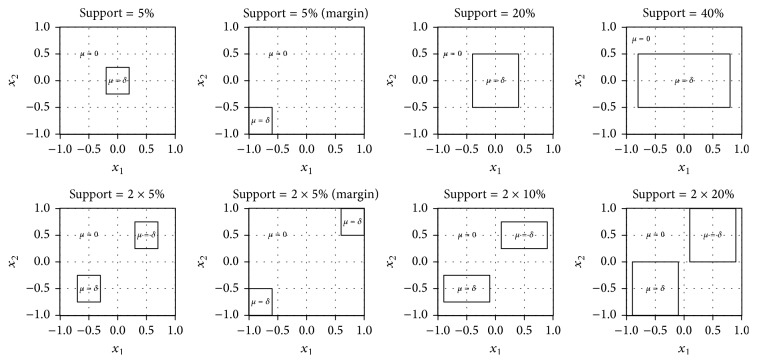
Designs of the simulations.

Boxes as shown in Figure 4 are defined by *X*_1_ and *X*_2_ only. The quantitative outcome *Y*, which should be distributed differently within and outside the boxes, is generated by a random sample from a normal distribution, so that

(11) $$\begin{array}{c} Y_{i}\sim N\left(\;\mu_{i},1\;\right)\;i=1,\ldots ,n, \end{array}$$

with

(12) $$\begin{array}{c} \mu_{i}=\left\{\begin{array}{cc} \delta & observation\;\;i\;\;lies\;\;inside\;\;a\;\;box\\ 0 & else. \end{array}\right. \end{array}$$

As shown in Figure 4, one or two boxes are used with different sizes. If there are two of them, they are equally sized with no overlapping, while same *δ* is applied in both. Their support takes the values 5%, 20%, 40%, 2 · 5%, 2 · 10%, and 2 · 20%, respectively. To explore the influence of the box's position on the results, situations were included with one/two box(es) lying at the margin of the distribution of the covariates. The higher the value of *δ* chosen, the larger the effect of the subgroup by a constant random noise over the groups (here, *σ*^2^ = 1). So *δ* determines the signal-to-noise ratio which in this case is *δ*/*σ* = *δ*. The simulations are performed for every *δ* in the sequence {0,0.33,0.67,1, 1.33,1.67,2, 2.33,2.67,3} and each simulation is repeated 250 times.

#### 4.1.2. Evaluation Criteria

To measure the ability of an algorithm to identify given subgroups, a criterion for the similarity of two classifications is needed. With this, it is possible to quantify the goodness of a prediction, made by one of the algorithms, by comparing its classification to the true one of the simulated data. This can be done via a cross table such as Table 1. Of primary interest is how many observations are allocated correctly (TP and TN) compared to those incorrectly allocated (FN and FP).

Two criteria that address this issue are sensitivity and specificity. These can be calculated as follows:

(13) Sens=TPTP+FN,Spec=TNTN+FP.

Sensitivity, which is also called true positive rate, describes the proportion of positive observations (i.e., belonging to the true subgroup) that are correctly identified as part of a subgroup by the algorithm. Specificity, or true negative rate, describes the proportion of negative observations correctly classified as not belonging to a subgroup. Both measurements have a range from 0 to 1 and they are only useful if they are considered together.

A closely related criterion that combines the sensitivity and specificity is Youden's J statistic (cf. [12]) which can be calculated as

(14) $$\begin{array}{c} J=Sens+Spec-1. \end{array}$$

This statistic weights sensitivity and specificity equally and is normalized so that it takes the value 0, on average, if the classification by the algorithm is completely random. It does not depend on the support size of the predicted subgroup. The value 1 in this case is taken if the two classifications are exactly the same. Due to this, Youden's J statistic is a suitable criterion to compare the agreement between the predicted classification and the true classification.

The estimation of sensitivity and specificity may be biased if performed on the training data. According to that, test data consisting of another 10,000 observations was drawn from the same data generating process in order to obtain unbiased estimates of sufficient precision [13].

It should be noted that all of the above-mentioned statistics are commonly used for the evaluation of diagnostic tests. However, they can appropriately be applied in the context of the identification of subgroups, as done, for instance, in [14].

#### 4.1.3. Settings of the Applied Functions

In this study, three different methods for the identification of subgroups were compared to each other, with two of them being variations of PRIM.

As described in Section 2.4, the user involvement of PRIM plays an important part which means that it is not possible to specify general rules for the application of PRIM. For that reason, two different approaches were followed, with the first one reflecting a user involvement that is optimal regarding the support sizes. This implies that the user knows the true subgroup sizes, which is an overoptimistic scenario in most cases. Careful investigation of trajectories could at least help to approximate this optimal result. In summary, this algorithm seeks for one or two boxes by maximizing the box mean over all boxes having at least the true support size.

The second variation of PRIM was to seek for boxes with the largest possible support for a given minimum box mean of *f*_min_ = 2. Since the true box mean *δ* ranges from 0 to 3, there are situations included in which the simulated user underestimates or overestimates the true box mean. This approach should represent a rather “bad” or naive user involvement, because the user always sticks to the same assumed *f*_min_ independent of the current situation (overall mean, trajectory, etc.).

These two approaches shall represent the extremes of possible user involvement. In reality, results would probably lie somewhere in between. *α* was set to {0.01,0.02,…, 0.5} each time. Bootstrap sampling was not performed to limit the computational effort needed and due to our experience that it is more important to process several *α* values instead. In both cases, the maximum number of boxes determined by PRIM was restricted to the actual number of true subgroups.

The third method is a version of CART. The R-function rpart() from the package rpart (cf. [11]) was used to implement CART. Since the outcome used was continuous, regression trees were fitted. When there are one or two true subgroups, the leaf with the highest or the two leafs with the highest mean outcome determine(s) the estimated subgroup(s). For a fair comparison and because the maximum number of boxes found by PRIM is restricted, the maximum depth of the trees in CART was also limited. This stops them from becoming unnecessarily complex. Therefore, the maximum depth of a tree (corresponding to the function parameter maxdepth) was set to 4 and 8 as required in the case of one or two true subsets, respectively. In the cases with the boxes lying at the margins, this parameter was set to 2 or 4.

A second version of CART was also implemented, where the maximum depth of the trees was not limited substantially with a value of 30. After the tree was fitted, it was pruned to minimize its cross-validated prediction error. This procedure is intended to mimic what applicants usually do.

The minimum support beta_min was set to 7/*n* for the second PRIM version (PRIM (*f*_min_ = 2)), since the default size of a leaf in rpart() is at least 7 observations. In the first version (PRIM (opt. *β*)), beta_min is already determined by the true support size.

#### 4.1.4. Results

**Figure 5:**
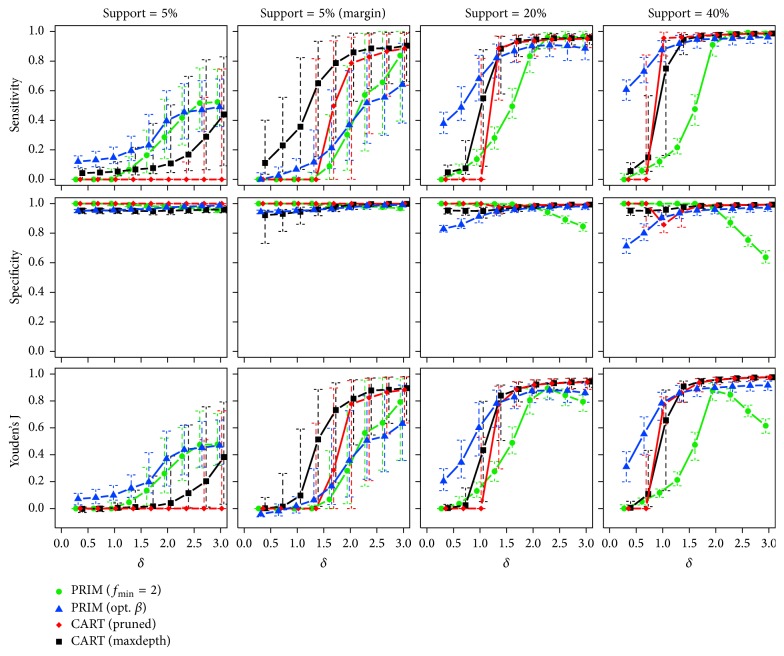
Medians and interquartile ranges of the sensitivities, specificities, and Youden's J statistics of all simulation runs with *n* = 250 observations and one true subgroup.

*One True Subgroup*.  Figure 5 plots the observed median sensitivity, specificity, and Youden's J statistic (14) of each method against the effect size *δ* for different support sizes in the case of a single true subgroup and an overall sample size of *n* = 250. Corresponding interquartile ranges of the 250 runs are shown by (dashed) error bars.

For a small centered subgroup with *β* = 5%, the specificity of all methods is high. This is easy to accomplish in such cases, even for algorithms that detect no subgroup, that is, miss the true subgroup. Therefore, the results for sensitivity should be focused upon. For each method, except for CART (pruned), the median sensitivity increases with rising effect sizes *δ*. PRIM (opt. *β*) benefits from the correct prior knowledge about the actual size of the subgroup and performs best. For *δ* ≥ 1.5 PRIM (*f*_min_ = 2) is on a similar level. Similar results on the sensitivity are observed for *β* = 20% and *β* = 40% with the important difference that CART shows a superior performance apart from small effect sizes. For PRIM (*f*_min_ = 2), there is a noticeable decrease of specificity for *δ* ≥ 2. The latter can be explained by the tendency of this method to select too big subgroups if the true subgroup has an actual mean that is larger than the one searched for. For PRIM (opt. *β*), the specificity for small *δ* is slightly lower. The reason is that it is forced by the input parameters to choose a subgroup with at least the true support size. All methods show a better performance for subgroups lying at the margin of the input space for given *β* of 5%. In this case, both CART methods seem to perform better than PRIM.

Table 2 lists the proportions of runs in which a subgroup was predicted by the methods. PRIM (opt. *β*) and CART (maxdepth), for all combinations of *δ* and the true support size *β*, find a subgroup in 100% of the cases. Even if in fact there is no subgroup, that is, *δ* = 0, both methods always predict one. Therefore, the methods show a false positive rate of 100% in such cases. At least for PRIM (opt. *β*), this result is not very surprising, because there was no constraint regarding the box mean, which makes the algorithm always find a subgroup with the specified support size. Only the methods PRIM (*f*_min_ = 2) and CART (pruned) do not always predict subgroups, which is why they have low false positive rates in case that there is no subgroup (*δ* = 0). The larger the true subgroup becomes, the more often the methods detect subgroups with a steeper increase for PRIM. In conjunction with the results about the sensitivity of methods (cf.  Figure 5), one can conclude that although the methods (almost) always find something, it is not until increased effect sizes that these findings show some concordance to the true subgroup.

So far, the case with one true subgroup and *n* = 250 observations has been presented. Results for *n* = 500 and *n* = 1000 are similar and are therefore shown in Appendix A. In general, all methods predict the subgroups better than for less observations, with CART showing the strongest improvement.

**Figure 6:**
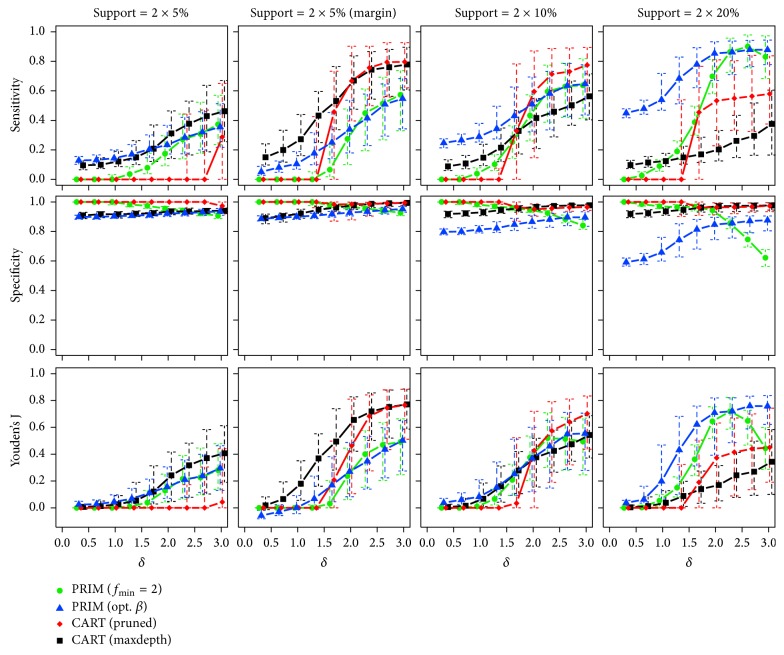
Medians and interquartile ranges of the sensitivities, specificities, and Youden's J statistics of all simulation runs with *n* = 250 observations and two true subgroups.

*Two True Subgroups*. Starting with the lowest sample size (*n* = 250), the observed medians and interquartile ranges of the corresponding sensitivity, specificity, and Youden's J statistic are illustrated in Figure 6. The proportions of runs with predicted subgroups are also listed in Table 2.

Independent of the effect size and for rising support of the true boxes, PRIM (opt. *β*) again benefits from the correct specification of the box sizes searched for and is always among the best performing approaches in terms of sensitivity, if the subgroups do not lie at the margin. PRIM (*f*_min_ = 2) can only catch up for higher values of the effect size, that is, when its specification about the searched effect becomes correct, too. The performance of CART decreases with increasing support sizes. This deficiency is possibly because of the well-known fact that the algorithm often fails to find a useful first split in chessboard-like “XOR” problems (cf. [15]). Switching the positions of the subgroups towards the margin of the input space makes both CART versions clearly improve. Referring to specificity, all methods show very good performances, while decreased values can be observed for PRIM (opt. *β*) and PRIM (*f*_min_ = 2) with low and high effect sizes, respectively. Similar results for increased sample sizes of *n* = 500 and *n* = 1000 are given in Appendix B. CART shows again the most pronounced improvements, here.

### 4.2. Application to Clinical Data

In this section, the application of PRIM and CART is illustrated using a real data example. The data set PimaIndiansDiabetes2 has been taken from the R-package mlbench (cf. [16]). It contains 768 observations from individuals that were tested “positive” or “negative” for diabetes. The data are from women with a minimum age of 21 and a Pima Indian heritage. From the 768 women, 268 (35%) tested positive and 500 (65%) tested negative. In addition to the outcome variable, the data set contains 8 quantitative covariates: pregnant (number of pregnancies), glucose (plasma glucose concentration (measured by a glucose tolerance test)), pressure (diastolic blood pressure [mmHg]), triceps (triceps skin fold thickness [mm]), insulin (2-hour serum insulin [mu U/mL]), mass (body mass index), pedigree (diabetes pedigree function), and age (age in years).

The aim of the analysis is to identify a possible association between the covariates and the occurrence of a positive test result which can be addressed by finding subgroups with proportionally many cases of diabetes.

There are some missing values that need to be handled in the analysis methods. Most of them can be found in the variables triceps and insulin with absolute (relative) frequencies of 227 (30%) and 374 (49%). Out of all 768 observations, there are only 392 (51%) complete cases, which draws the appropriateness of complete case analysis into question in this case.

**Figure 7:**
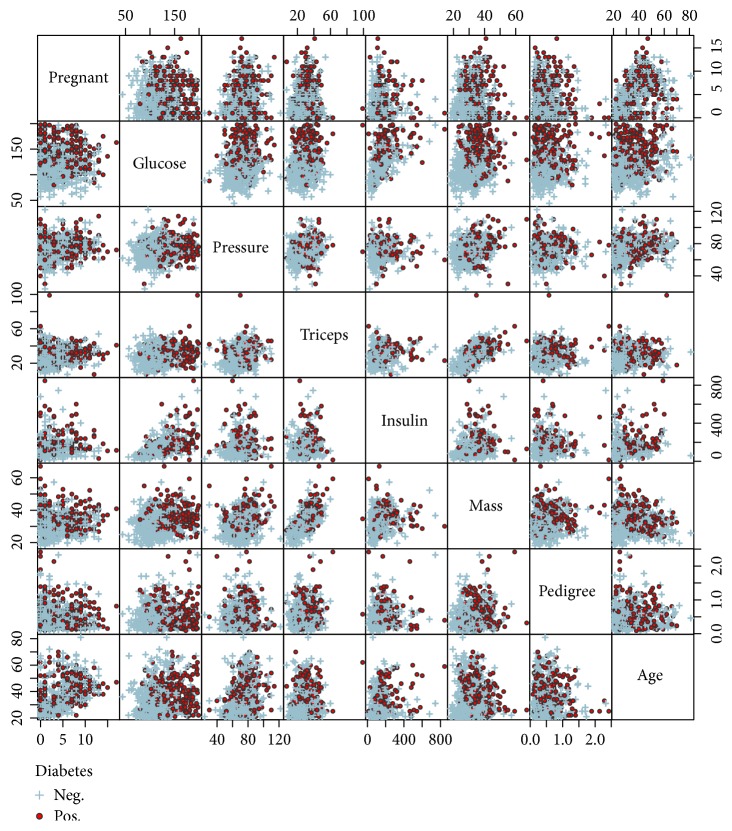
Graphical illustration of the diabetes data by pairwise scatter plots.

The data are illustrated in Figure 7 by pairwise scatter plots of all covariates. This figure gives a first impression of how the variables are distributed and their pairwise correlations. For example, there appears to be a quite strong positive correlation between triceps and mass along with some other medium and weak correlations. Relations to the outcome can be derived too and point at potential candidates for a splitting criterion. It seems that women with high glucose and mass (BMI) values are more likely to have diabetes.

**Figure 8:**
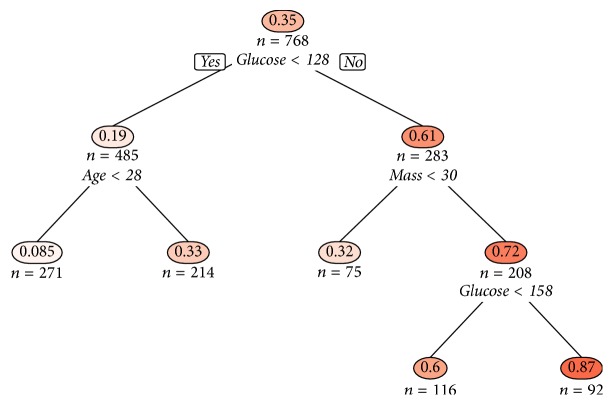
Result of CART applied on the diabetes data illustrated as a decision tree. For each node, the proportions of positive cases in this group and the number of contained observations are shown.

A classification tree (cf.  Section 3) was fit to the data using the function rpart() with its default settings. The tree was pruned according to the 1-SE rule (cf. [17]). The resulting decision tree is illustrated in Figure 8. Missing values are handled by CART internally via surrogate splits (cf. [18]). The suggestion from Figure 7 that the variables glucose and mass can split the data well is confirmed by the tree, where these variables are also used for splitting rules. age also has predictive value in this model.

Since the aim is to find a subgroup with proportionally many cases of diabetes, the leaf with the highest mean outcome can be seen as this subgroup by CART. So the high risk group defined by CART, which can also be seen as a box *B*_CART_ containing 92 (12%) observations, has a mean outcome of 0.87 and is defined as

(15) $$\begin{array}{c} x\in B_{CART}=\left\{\begin{array}{cc} glucose>158, & \\ mass>30. & \end{array}\right. \end{array}$$

PRIM was applied once using singular peeling without bootstrapping and *α* = 0.05 and once using multiple peeling with *s* = 10 bootstrap samples and the **α**-vector (0.01,0.02,…, 0.5). It can also handle missing values in the covariates if applied as suggested by Friedman and Fisher [5]. In this case, all missing values in one covariate are treated as a category, so that in each peeling and pasting step this whole category can be peeled or pasted from the current box. This way, the algorithm tends to use surrogate variables instead of variables with many missing values. If the category that indicates missing values is used for the box definition, this suggests that the data may not be missing completely at random.

**Figure 9:**
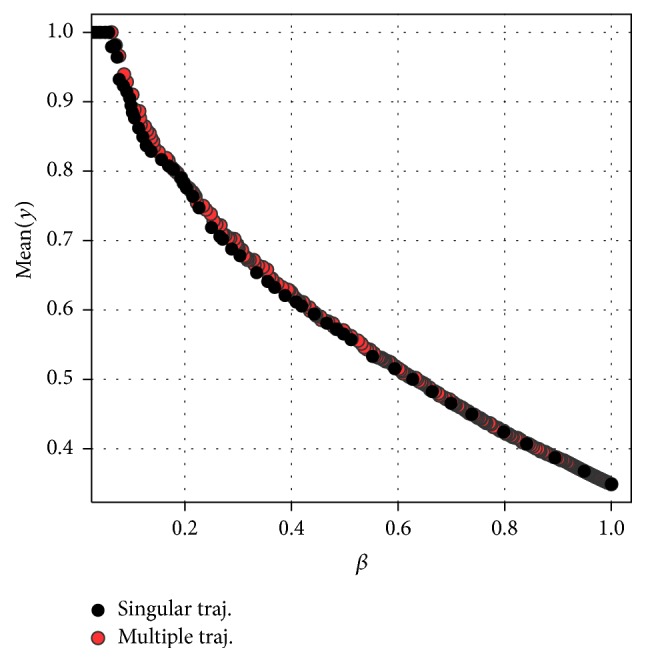
Trajectories for singular and multiple peeling (after removal of the dominated boxes) on the diabetes data.

The trajectories are shown in Figure 9, where for multiple peeling all dominated boxes were removed. Multiple peeling seems to provide only small improvement over the singular version here. Both trajectories are quite smooth, such that they do not suggest a definite box for selection. A user would have to make a deliberate decision based on subject specific knowledge. This flexibility is a desirable property of PRIM and is seldom given by other methods.

If the aim was, for instance, to search for a subgroup with a proportion of positive tested women of at least 80% and maximum support (by using the multiple trajectory), the resulting box *B*^(1)^ which can be seen as a high risk group would be defined as

(16) $$\begin{array}{c} x\in B^{\left(1\right)}=\left\{\begin{array}{cc} glucose>129,triceps>15, & \\ 126<insulin<544, & \\ mass>30,mass\neq missing, & \\ age>24. & \end{array}\right. \end{array}$$

Again, the variables glucose, mass (BMI), and age, which also played an important role in the CART model, are used. In addition, the variables triceps and insulin define further box limits. Concerning BMI, missing values are excluded from the box. This could indicate a relation between the probability of a value to be missing and the outcome.

By this simple box definition, the data can be divided into a subgroup with a very high mean outcome (0.8) containing 140 (18%) observations and a group that contains the remainder of observations with a relatively small mean (0.25). With the covering procedure, even more boxes can be sought. This would lead to the identification of three more boxes with means 0.81, 0.83, and 0.83 containing 37 (5%), 29 (4%), and 29 (4%) observations, respectively. The remaining 533 observations have a proportion of positive diabetes tests of approximately 15%.

## 5. An Extension of PRIM for Survival Data

As described above, the original PRIM algorithm can only handle quantitative and binary (0/1 coded) outcomes. A useful extension, especially in the medical domain, is to enable PRIM to handle censored survival outcomes. In such cases, every observation provides a survival time *t*_*i*_ and an indicator *δ*_*i*_ taking the value 1 if the event occurred at *t*_*i*_ and 0 if the observation is censored. A suggested extension of PRIM is to use the hazard rate as the target function for maximization.

(17) $$\begin{array}{c} f\left(\;t,\delta \;\right)=\frac{\sum_{i=1}^{n}\delta_{i}}{\sum_{i=1}^{n}t_{i}}. \end{array}$$

Under the assumption of time-constant risks, subgroups with different survival can be sought with this target function.

*Application Example*. To illustrate the application of PRIM on censored survival data, the data set “Whitehall 1” from [19] was used. It is from a prospective, cross-sectional cohort study of 17260 male British Civil Servants employed in London. The aim of this study was to examine the influence of some baseline variables on the risk of dying due to a coronary heart disease (CHD). Therefore, the time to death from CHD was measured for the participants as a censored survival time. Additionally, the following variables were measured: cigs (daily cigarette consumption), map (mean arterial pressure), age (age (years)), ht (height (cm)), wt (weight (kg)), chol (cholesterol (mmol/L)), and jobgrade (job grade (nominal)).

To find subgroups with high risk of dying from CHD, PRIM was applied with the hazard rate as target function by using multiple peeling with *s* = 5, **α** = (0.01,0.03,0.05,…, 0.31), and *β*_0_ = 0.01. Since the not dominated boxes of the multiple trajectory form a smooth curve, the user can practically choose every box out of these. So the proportion of box definitions in which a variable is included can be interpreted as the probability of this variable to define the subgroup, if the user chooses randomly out of these boxes.

In this data example, we get lower boundaries for the variables age, map, chol, and cigs in 99%, 80%, 31%, and 17% of the relevant boxes, which indicates that increases in those variables are associated with increasing risk of CHD. This result is similar to the one reported in [20] (p. 142), where the authors used fractional polynomials with logistic regression to model the 10-year survival rate and they concluded that increases in age, cigarette consumption, cholesterol, body weight, and mean arterial pressure are associated with increasing risk of CHD and the opposite is true for height.

## 6. Discussion and Conclusion

PRIM, as described in Section 2, is a very flexible tool for the identification of areas in the data which show increased or decreased outcome values. Besides PRIM, there are other methods pursuing similar goals with different strategies, such as CART.

In a simulation study, both methods showed strengths and weaknesses. PRIM seemed to be the better choice in several rather complex data settings with small subgroups, few observations, and small effect sizes. In all other cases, CART was a competitive alternative and showed advantages in rather simple settings. This differential behaviour makes it difficult to give a universal rule about which method should be preferred, especially as the complexity of the problem is usually unknown to the applicant.

PRIM has high user involvement (see Section 2.4), which can strongly influence the goodness of the result. Misspecification of the subgroup properties, that is, mean outcome and size, can substantially decrease the performance. This also became clear in the simulation study, where two different versions of PRIM were applied simulating different acting users. These two versions in some cases (especially in simpler data settings) differed strongly. This fact underlines the importance of a close interaction between a user and the PRIM algorithm, for example, by looking at the trajectories to obtain a suitable result.

A real data example showed how these two methods can be applied for subgroup identification. Here, both methods came to a similar result. It again became clear that PRIM can be flexibly tuned by the users concerning their needs, whereas CART, although simpler to use, is rather static.

*R-Implementation*. All features of PRIM described in this paper and some more are implemented in the R-package PRIM, which is available at GitHub (https://github.com/ao90/PRIM) together with a manual documenting its functions. The package contains additional functions for graphical diagnostics and other features described in [5].

## Supplementary Material

Appendix C: R-code for creating the figures of the manuscript.
Appendix D: R-code for applying the simulations of section 4.1.
Appendix E: R-code for the illustration of the simulation results of section 4.1.
Appendix F: R-code of the diabetes data example of section 4.2.
Appendix G: R-code of the whitehall data example of section 5.

## Figures and Tables

**Figure 10:**
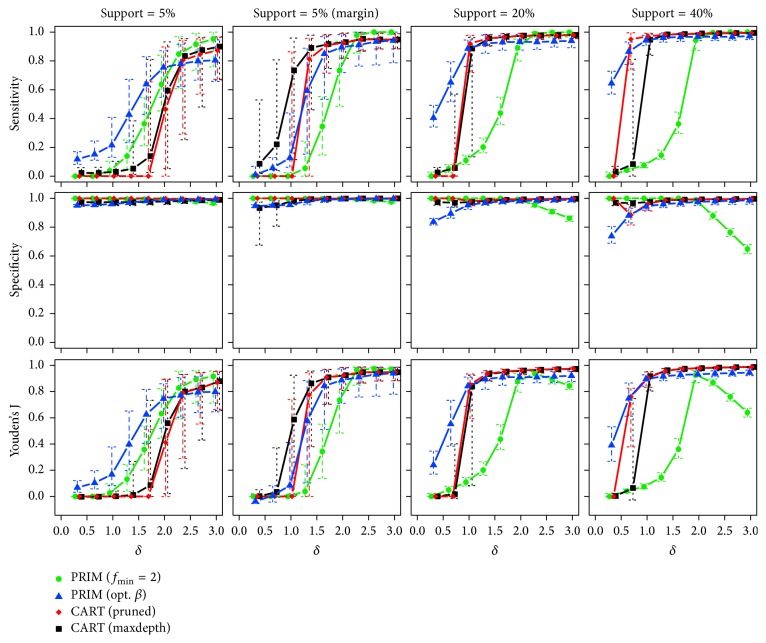
Medians and interquartile ranges of the sensitivities, specificities, and Youden's J statistics of all simulation runs with *n* = 500 observations and one true subgroup.

**Figure 11:**
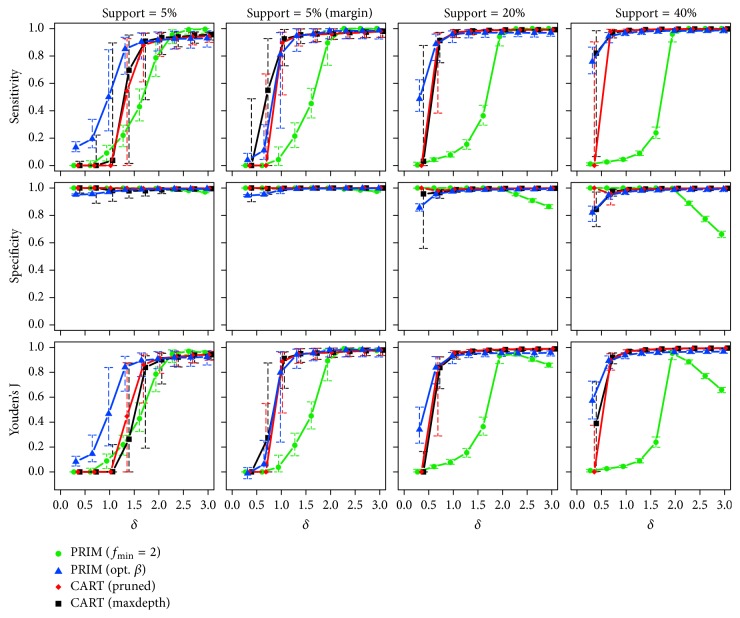
Medians and interquartile ranges of the sensitivities, specificities, and Youden's J statistics of all simulation runs with *n* = 1000 observations and one true subgroup.

**Figure 12:**
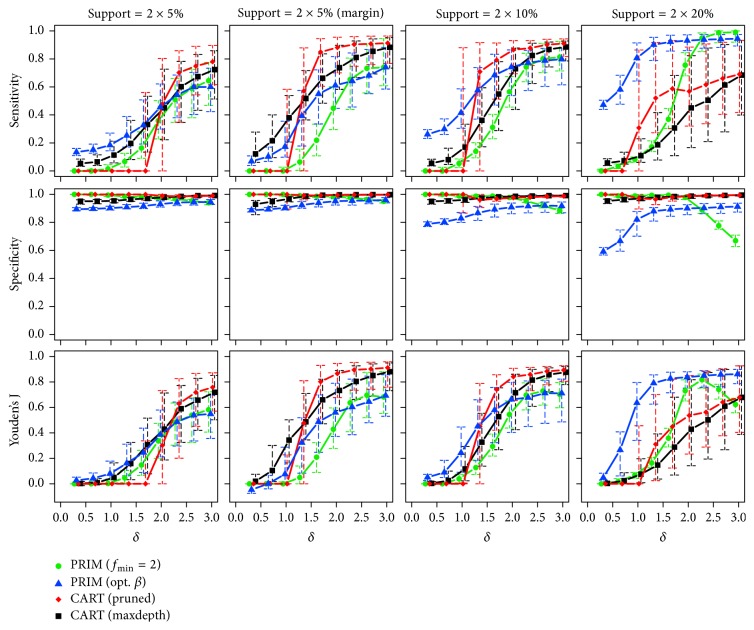
Medians and interquartile ranges of the sensitivities, specificities, and Youden's J statistics of all simulation runs with *n* = 500 observations and two true subgroups.

**Figure 13:**
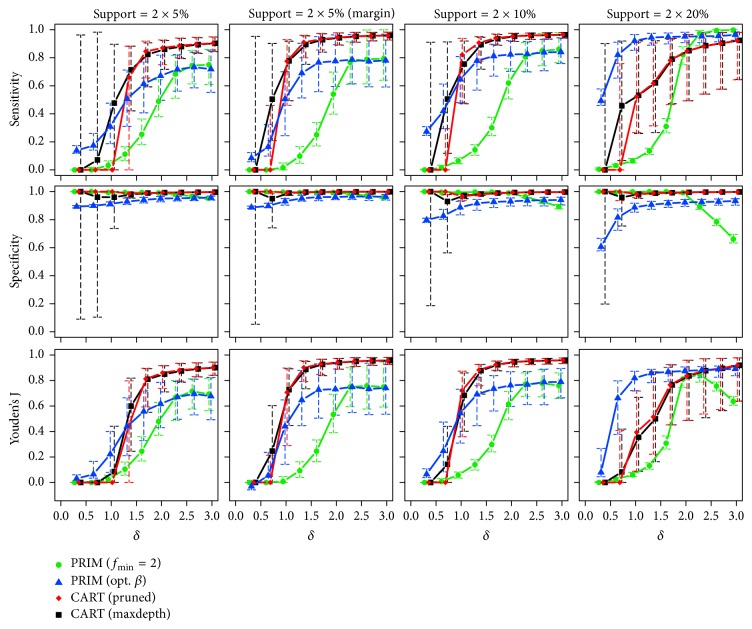
Medians and interquartile ranges of the sensitivities, specificities, and Youden's J statistics of all simulation runs with *n* = 1000 observations and two true subgroups.

**Table 1:** Cross table of true against predicted classification (1 = observation belongs to the subgroup according to the corresponding classification; 0 = otherwise).

		Classification of the algorithm	
		1	0
True	1	True positives (TP)	False negatives (FN)
Classification	0	False positives (FP)	True negatives (TN)

**Table 2:** Proportions of cases with a predicted subgroup when using the methods PRIM (*f*_min_ = 2) and CART (pruned) for given *n* = 250 observations and one or two true subgroups. Results for the methods PRIM (opt. *β*) and CART (maxdepth) are not shown here, because their proportions were 1 for each *β* and *δ*.

*β*	Method	0	0.33	0.67	1	1.33	1.67	2	2.33	2.67	3
5%	PRIM (*f*_min_ = 2)	0.05	0.07	0.14	0.32	0.55	0.76	0.9	0.98	1	1
	CART (pruned)	0.03	0.04	0.04	0.05	0.18	0.18	0.2	0.27	0.37	0.47

5% (margin)	PRIM (*f*_min_ = 2)	0.08	0.08	0.09	0.21	0.4	0.69	0.86	0.93	0.98	0.99
	CART (pruned)	0.08	0.06	0.08	0.13	0.41	0.58	0.69	0.77	0.86	0.92

20%	PRIM (*f*_min_ = 2)	0.1	0.2	0.58	0.9	0.99	1	1	1	1	1
	CART (pruned)	0.04	0.06	0.15	0.41	0.85	0.97	1	1	1	1

40%	PRIM (*f*_min_ = 2)	0.11	0.34	0.82	1	1	1	1	1	1	1
	CART (pruned)	0.08	0.1	0.35	0.91	1	1	1	1	1	1

2 × 5%	PRIM (*f*_min_ = 2)	0.08	0.08	0.14	0.36	0.63	0.86	0.97	1	1	1
	CART(pruned)	0.17	0.19	0.06	0.08	0.11	0.16	0.24	0.31	0.4	0.54

2 × 5% (margin)	PRIM (*f*_min_ = 2)	0.05	0.07	0.11	0.25	0.5	0.82	0.97	1	1	1
	CART (pruned)	0.2	0.21	0.08	0.13	0.3	0.61	0.8	0.9	0.95	0.97

2 × 10%	PRIM (*f*_min_ = 2)	0.06	0.15	0.34	0.71	0.93	1	1	1	1	1
	CART (pruned)	0.14	0.13	0.03	0.08	0.25	0.56	0.76	0.86	0.92	0.96

2 × 20%	PRIM (*f*_min_ = 2)	0.08	0.24	0.67	0.97	1	1	1	1	1	1
	CART (pruned)	0.16	0.17	0.08	0.18	0.42	0.69	0.81	0.89	0.92	0.96

**Table 3:** Proportions of cases with predicted subgroups when using one of the methods for *n* = 500 observations and one true subgroup.

*β*	Method	0	0.33	0.67	1	1.33	1.67	2	2.33	2.67	3
5%	PRIM (*f*_min_ = 2)	0.1	0.13	0.28	0.59	0.86	0.99	1	1	1	1
	PRIM (opt. *β*)	1	1	1	1	1	1	1	1	1	1
	CART (pruned)	0.04	0.04	0.05	0.16	0.22	0.34	0.58	0.8	0.9	0.94
	CART (maxdepth)	1	1	1	1	1	1	1	1	1	1

5% (margin)	PRIM (*f*_min_ = 2)	0.08	0.11	0.16	0.35	0.64	0.93	1	1	1	1
	PRIM (opt. *β*)	1	1	1	1	1	1	1	1	1	1
	CART (pruned)	0.05	0.04	0.1	0.39	0.69	0.88	0.98	0.98	1	1
	CART (maxdepth)	0.98	0.99	0.99	0.99	1	1	1	1	1	1

20%	PRIM (*f*_min_ = 2)	0.1	0.34	0.78	0.99	1	1	1	1	1	1
	PRIM (opt. *β*)	1	1	1	1	1	1	1	1	1	1
	CART (pruned)	0.04	0.06	0.23	0.92	1	1	1	1	1	1
	CART (maxdepth)	1	1	1	1	1	1	1	1	1	1

40%	PRIM (*f*_min_ = 2)	0.1	0.46	0.97	1	1	1	1	1	1	1
	PRIM (opt. *β*)	1	1	1	1	1	1	1	1	1	1
	CART (pruned)	0.06	0.13	0.85	1	1	1	1	1	1	1
	CART (maxdepth)	1	1	1	1	1	1	1	1	1	1

**Table 4:** Proportions of cases with predicted subgroups when using one of the methods for *n* = 1000 observations and one true subgroup.

*β*	Method	0	0.33	0.67	1	1.33	1.67	2	2.33	2.67	3
5%	PRIM (*f*_min_ = 2)	0.2	0.24	0.43	0.86	0.98	1	1	1	1	1
	PRIM (opt. *β*)	1	1	1	1	1	1	1	1	1	1
	CART (pruned)	0.04	0.04	0.04	0.19	0.61	0.87	0.97	1	1	1
	CART (maxdepth)	0.38	0.38	0.48	0.66	0.84	0.94	0.97	0.98	1	1

5% (margin)	PRIM (*f*_min_ = 2)	0.19	0.21	0.34	0.72	0.96	1	1	1	1	1
	PRIM (opt. *β*)	1	1	1	1	1	1	1	1	1	1
	CART (pruned)	0.07	0.11	0.31	0.82	0.99	1	1	1	1	1
	CART (maxdepth)	0.31	0.44	0.72	0.94	1	1	1	1	1	1

20%	PRIM (*f*_min_ = 2)	0.13	0.5	0.97	1	1	1	1	1	1	1
	PRIM (opt. *β*)	1	1	1	1	1	1	1	1	1	1
	CART (pruned)	0.06	0.14	0.85	1	1	1	1	1	1	1
	CART (maxdepth)	0.42	0.66	0.99	1	1	1	1	1	1	1

40%	PRIM (*f*_min_ = 2)	0.12	0.63	1	1	1	1	1	1	1	1
	PRIM (opt. *β*)	1	1	1	1	1	1	1	1	1	1
	CART (pruned)	0.07	0.36	1	1	1	1	1	1	1	1
	CART (maxdepth)	0.42	0.9	1	1	1	1	1	1	1	1

**Table 5:** Proportions of cases with predicted subgroups when using one of the methods for *n* = 500 observations and two true subgroups.

*β*	Method	0	0.33	0.67	1	1.33	1.67	2	2.33	2.67	3
2 × 5%	PRIM (*f*_min_ = 2)	0.14	0.18	0.32	0.6	0.9	0.98	1	1	1	1
	PRIM (opt. *β*)	1	1	1	1	1	1	1	1	1	1
	CART (pruned)	0.08	0.06	0.2	0.16	0.19	0.42	0.64	0.82	0.94	0.98
	CART (maxdepth)	1	1	1	1	1	1	1	1	1	1

2 × 5% (margin)	PRIM (*f*_min_ = 2)	0.1	0.12	0.2	0.48	0.77	0.98	1	1	1	1
	PRIM (opt. *β*)	1	1	1	1	1	1	1	1	1	1
	CART (pruned)	0.04	0.04	0.21	0.4	0.74	0.94	1	1	1	1
	CART (maxdepth)	0.99	0.99	1	1	1	1	1	1	1	1

2 × 10%	PRIM (*f*_min_ = 2)	0.1	0.18	0.48	0.86	1	1	1	1	1	1
	PRIM (opt. *β*)	1	1	1	1	1	1	1	1	1	1
	CART (pruned)	0.05	0.07	0.19	0.37	0.74	0.94	0.99	1	1	1
	CART (maxdepth)	1	1	1	1	1	1	1	1	1	1

2 × 20%	PRIM (*f*_min_ = 2)	0.1	0.33	0.86	1	1	1	1	1	1	1
	PRIM (opt. *β*)	1	1	1	1	1	1	1	1	1	1
	CART (pruned)	0.04	0.04	0.24	0.59	0.87	0.98	0.99	1	1	1
	CART (maxdepth)	1	1	1	1	1	1	1	1	1	1

**Table 6:** Proportions of cases with predicted subgroups when using one of the methods for *n* = 1000 observations and two true subgroups.

*β*	Method	0	0.33	0.67	1	1.33	1.67	2	2.33	2.67	3
2 × 5%	PRIM (*f*_min_ = 2)	0.19	0.25	0.46	0.82	0.98	1	1	1	1	1
	PRIM (opt. *β*)	1	1	1	1	1	1	1	1	1	1
	CART (pruned)	0.11	0.04	0.08	0.23	0.71	0.98	1	1	1	1
	CART (maxdepth)	0.37	0.38	0.53	0.74	0.92	0.98	1	1	1	1

2 × 5% (margin)	PRIM (*f*_min_ = 2)	0.15	0.22	0.34	0.71	0.96	1	1	1	1	1
	PRIM (opt. *β*)	1	1	1	1	1	1	1	1	1	1
	CART (pruned)	0.1	0.08	0.3	0.86	0.99	1	1	1	1	1
	CART (maxdepth)	0.36	0.46	0.82	0.97	1	1	1	1	1	1

2 × 10%	PRIM (*f*_min_ = 2)	0.14	0.29	0.75	0.97	1	1	1	1	1	1
	PRIM (opt. *β*)	1	1	1	1	1	1	1	1	1	1
	CART (pruned)	0.11	0.06	0.26	0.88	1	1	1	1	1	1
	CART (maxdepth)	0.37	0.46	0.8	0.97	1	1	1	1	1	1

2 × 20%	PRIM (*f*_min_ = 2)	0.16	0.56	0.94	1	1	1	1	1	1	1
	PRIM (opt. *β*)	1	1	1	1	1	1	1	1	1	1
	CART (pruned)	0.08	0.05	0.5	0.93	1	1	1	1	1	1
	CART (maxdepth)	0.36	0.44	0.78	0.96	1	1	1	1	1	1

## Appendix

### A. Further Simulation Results with One True Subgroup

See Figures 10 and 11 and Tables 3 and 4.

### B. Further Simulation Results with Two True Subgroups

See Figures 12 and 13 and Tables 5 and 6.
