# Supplementary material for: Nonparametric Subgroup Identification by PRIM and CART: A Simulation and Application Study
Source: Comput Math Methods Med. 2017 May 22;2017:5271091. doi: 10.1155/2017/5271091 (PMC5458436; doi:10.1155/2017/5271091)
Supplement: Supplementary file 1 — Appendix C: R-code for creating the figures of the manuscript. Appendix D: R-code for applying the simulations of section 4.1. Appendix E: R-code for the illustration of the simulation results of section 4.1. Appendix F: R-code of the diabetes data example of section 4.2. Appendix G: R-code of the whitehall data example of section 5. [file 5271091.f1.pdf]

Appendix C

```
# choose path for saving the images
# setwd("yourpath")

### install package PRIM (can be skipped if already done)
#install.packages("devtools")
#library(devtools)
#install_github("ao90/PRIM")

library(PRIM)


###############
### Peeling ###
###############

# generate random data
set.seed(123)
n <- 1000
x1 <- runif(n = n, min = -1)
x2 <- runif(n = n, min = -1)
y <- ifelse(x1>0 & x1<0.5 & x2>-.8 & x2<.1, sample(0:1, size = n, prob = c(.1,.9), replace = T), sample(0:1, size = n, prob =
c(.9,.1), replace = T))
x1 <- x1*10
x2 <- x2*10
x3 <- sample(c("a", "b", "c"), size = n, replace = T)

# Illustration of the box sequence in the peeling algorithm
prim <- PRIM_peel(data=cbind(y,x1,x2), peel_alpha = .25, beta_min = .075) # apply peeling to random data
postscript("peel_seq.eps", width=7.5, height=7.5, horizontal=F, paper="special")
par(mar=c(0,0,0,0),xpd=NA, mfrow=c(2,2), oma=c(4,4,3,5))
plot(x1,x2,col=ifelse(y==0, "lightblue", "grey20"), pch=ifelse(y==0, 3, 21), bg=adjustcolor("red", offset = c(0, .35, .35, .35)),
las=1, xlab="", xaxt="n", ylab=expression("x"[2]), cex.lab=1.2, cex=.8)
for(it in 1){
  prim$box[it,]<-ifelse(prim$box[it,]==-Inf, -10, ifelse(prim$box[it,]==Inf, 10, prim$box[it,]))
  polygon(x=c(prim$box[it,3],prim$box[it,1],prim$box[it,1], prim$box[it,3]),
        y=c(prim$box[it,4], prim$box[it,4], prim$box[it,2], prim$box[it,2]), lwd=2)
}
q1<-quantile(x1, probs = c(0,.25, .75,1))
q2<-quantile(x2, probs = c(0,.25, .75,1))
segments(x0=c(q1[1], q1[1], q1[2],q1[3]), x1=c(q1[4], q1[4], q1[2],q1[3]), y0=c(q2[2], q2[3], q2[1], q2[1]), y1=c(q2[2], q2[3],
q2[4], q2[4]), lty=c("dashed", "dashed","dotted", "dotted"), lwd=1.5)

text(x = 0, y = 0, labels = expression("B"[1]), cex = 3)
text(x = -7.5, y = 0, labels = expression("b"["11-"]), cex = 2.5)
text(x = 7.5, y = 0, labels = expression("b"["11+"]), cex = 2.5)
text(x = 0, y = -7.5, labels = expression("b"["12-"]), cex = 2.5)
text(x = 0, y = 7.5, labels = expression("b"["12+"]), cex = 2.5)

plot(x1,x2,col=ifelse(y==0, "lightblue", "grey20"), pch=ifelse(y==0, 3, 21), bg=adjustcolor("red", offset = c(0, .35, .35, .35)),
las=1, xlab="", ylab="", xaxt="n", yaxt="n", cex.lab=1.2, cex=.8)
for(it in 1:2){
  prim$box[it,]<-ifelse(prim$box[it,]==-Inf, -10, ifelse(prim$box[it,]==Inf, 10, prim$box[it,]))
  polygon(x=c(prim$box[it,3],prim$box[it,1],prim$box[it,1], prim$box[it,3]),
        y=c(prim$box[it,4], prim$box[it,4], prim$box[it,2], prim$box[it,2]), lwd=2)
}
text(x = 7.5, y = 0, labels = expression("b"[1]^"*"), cex = 2.5)
text(x = -2.5, y = 0, labels = expression("B"[2]), cex = 3)
legend("topright", inset=c(-0.225,0), legend = c("y = 1", "y = 0"), col = c("grey20", "lightblue"), pch = c(21,3),
pt.bg=adjustcolor("red", offset = c(0, .35, .35, .35)))
plot(x1,x2,col=ifelse(y==0, "lightblue", "grey20"), pch=ifelse(y==0, 3, 21), bg=adjustcolor("red", offset = c(0, .35, .35, .35)),
las=1, xlab=expression("x"[1]), ylab=expression("x"[2]), cex.lab=1.2, cex=.8)
for(it in 1:3){
  prim$box[it,]<-ifelse(prim$box[it,]==-Inf, -10, ifelse(prim$box[it,]==Inf, 10, prim$box[it,]))
```

```r
  polygon(x=c(prim$box[it,3],prim$box[it,1],prim$box[it,1], prim$box[it,3]),
         y=c(prim$box[it,4], prim$box[it,4], prim$box[it,2], prim$box[it,2]), lwd=2)
}
text(x = 7.5, y = 0, labels = expression("b"[1]^"*"), cex = 2.5)
text(x =-8, y = 0, labels = expression("b"[2]^"*"), cex = 2.5)
text(x = -1, y = 0, labels = expression("B"[3]), cex = 3)
plot(x1,x2,col=ifelse(y==0, "lightblue", "grey20"), pch=ifelse(y==0, 3, 21), bg=adjustcolor("red", offset = c(0, .35, .35, .35)),
las=1, xlab=expression("x"[1]), ylab="", yaxt="n", cex.lab=1.2, cex=.8)
for(it in 1:9){
prim$box[it,]<-ifelse(prim$box[it,]==-Inf, -10, ifelse(prim$box[it,]==Inf, 10, prim$box[it,]))
polygon(x=c(prim$box[it,3],prim$box[it,1],prim$box[it,1], prim$box[it,3]),
       y=c(prim$box[it,4], prim$box[it,4], prim$box[it,2], prim$box[it,2]), lwd=2)
}
text(x = 7.5, y = 0, labels = expression("b"[1]^"*"), cex = 2.5)
text(x =-8, y = 0, labels = expression("b"[2]^"*"), cex = 2.5)
text(x =-5, y = 0, labels = expression("b"[3]^"*"), cex = 2.5)
text(x =1, y = 7.5, labels = expression("b"[4]^"*"), cex = 2.5)
text(x =1, y = 2.75, labels = expression("b"[5]^"*"), cex = 2.5)
text(x =-2.3, y = -4.5, labels = expression("b"[6]^"*"), cex = 1.75)
text(x =2, y = -8.75, labels = expression("b"[7]^"*"), cex = 1.75)
text(x =-0.5, y = -3.2, labels = expression("b"[8]^"*"), cex = 1.55)
text(x =2.6, y = -3.2, labels = expression("B"[9]), cex = 3)
dev.off()


# Illustration of multiple and singular trajectory in one image
prim <- PRIM_peel(data=cbind(y, x1, x2), peel_alpha = .25, beta_min = 0) # apply singular peeling to random data
dat <- cbind.data.frame(y, x1, x2)
p <- PRIM_peel_bs(y~x1+x2, dat, seed = 123 , B = 10, peel_alpha=c(.01, .05, .1, .2)) # apply multiple peeling to random data
p_nd <- remove_dominated(p) # remove dominated boxes

postscript("mult_traj.eps", width=7.5, height=7.5, horizontal=F, paper="special")
plot(p$f~p$beta, ylab="f(y)", xlab=expression(beta), las=1, pch=21, ylim=c(0,1), xlim=c(0,1), col=0, xpd=NA)
points(p$f~p$beta, pch=16, cex=0.5, col="lightblue")
points(p_nd$f~p_nd$beta, pch=21, bg=adjustcolor("red", offset=c(0,.2,.2,.2)), col="grey20", cex=2)
points(prim$f~prim$beta, pch=16, col=1, cex=2)
legend("topright", legend = c("singular traj.", "multiple traj. (relevant boxes)", "multiple traj. (dominated boxes)"), pch =
c(16, 21, 16), pt.cex = c(2,2,.5), col=c(1, "grey20", "lightblue"), bty = "n",pt.bg=adjustcolor("red", offset=c(0,.2,.2,.2)))
abline(v=0.075, lty="dotted")
mtext(expression(beta[0]), side = 1, at = .075, line=1)
dev.off()


################
### Covering ###
################

# generate new random data
set.seed(123)
n <- 1000
x1 <- runif(n = n, min = -1)
x2 <- runif(n = n, min = -1)
y <- ifelse((x1>0 & x1<0.5 & x2>-.8 & x2<.1)|(x1>-.8 & x1<(-.4) & x2>0 & x2<.5), sample(0:1, size = n, prob = c(.1,.9), replace
= T), sample(0:1, size = n, prob = c(.9,.1), replace = T))
x1 <- x1*10
x2 <- x2*10

# apply PRIM
p <- PRIM(y ~ x1 + x2, data=cbind.data.frame(y, x1, x2), peel_alpha = .05, beta_min = .01, B = 0, f_min = .9)

# Illustration of the covering procedure
postscript("covering.eps", width=10, height=5, horizontal = F, paper = "special")
par(mar=c(0,0,0,0),xpd=NA, mfrow=c(1,2), oma=c(4,5,2,6))
plot(x1,x2,col=ifelse(y==0, "lightblue", "grey20"), pch=ifelse(y==0, 3, 21), bg=adjustcolor("red", offset = c(0, .35, .35, .35)),
las=1, xlab=expression("x"[1]), ylab=expression("x"[2]), cex.lab=1.2, cex=.8)
```

```
it<-1
polygon(x=c(p$box[it,3],p$box[it,1],p$box[it,1], p$box[it,3]),
       y=c(p$box[it,4], p$box[it,4], p$box[it,2], p$box[it,2]), lwd=2)
text(x = 2.5, y = -3.5, labels = expression("B"^{(1)}), cex = 2.5)
plot(x1[p$subsets[[3]]], x2[p$subsets[[3]]], col = ifelse(y[p$subsets[[3]]]==0, "lightblue" , "grey20"),
pch=ifelse(y[p$subsets[[3]]]==0, 3, 21), las=1, xlab=expression("x"[1]), ylab="", yaxt="n", cex.lab=1.2, cex=.8,
bg=adjustcolor("red", offset = c(0, .35, .35, .35)))
points(x1[p$subsets[[2]]], x2[p$subsets[[2]]], col = ifelse(y[p$subsets[[2]]]==0, "lightblue" , "grey20"),
pch=ifelse(y[p$subsets[[2]]]==0, 3, 21), cex=.8, bg=adjustcolor("red", offset = c(0, .35, .35, .35)))
it<-2
polygon(x=c(p$box[it,3],p$box[it,1],p$box[it,1], p$box[it,3]),
       y=c(p$box[it,4], p$box[it,4], p$box[it,2], p$box[it,2]), lwd=2)
text(x = -6, y = 3, labels = expression("B"^{(2)}), cex = 2.5)
legend("topright", inset=c(-0.205,0), legend = c("y = 1", "y = 0"), col = c("grey20", "lightblue"), pch = c(21,3),
pt.bg=adjustcolor("red", offset = c(0, .35, .35, .35)))
dev.off()


##########################
### Simulation designs ###
##########################

postscript("sim_des.eps", width=10, height=10/4*2, horizontal = F, paper = "special")
par(mfrow=c(2,4), mar=c(4, 4, 2, 1) + 0.1)

# 1group
# 5%
plot(NA, xlim=c(-.93,.93), ylim=c(-.93,.93), xlab = expression(x[1]), ylab = expression(x[2]), las=1)
mtext("support = 5%", 3, 0.5)
grid(col="grey60")
rect(xleft=-.2, ybottom = -.25, xright = .2, ytop = .25)
text(0,0, expression(paste(mu," = ",delta)))
text(-.5,.5, expression(paste(mu," = ",0)))

# 5% (margin)
plot(NA, xlim=c(-.93,.93), ylim=c(-.93,.93), xlab = expression(x[1]), ylab = expression(x[2]), las=1)
grid(col="grey60")
mtext("support = 5% (margin)", 3, 0.5)
rect(xleft=-1, ybottom = -1, xright = -.6, ytop = -.5)
text(-.8,-.75, expression(paste(mu," = ",delta)))
text(-.5,.5, expression(paste(mu," = ",0)))

# 20%
plot(NA, xlim=c(-.93,.93), ylim=c(-.93,.93), xlab = expression(x[1]), ylab = expression(x[2]), las=1)
grid(col="grey60")
mtext("support = 20%", 3, 0.5)
rect(xleft=-.4, ybottom = -.5, xright = .4, ytop = .5)
text(0,0, expression(paste(mu," = ",delta)))
text(-.75,.5, expression(paste(mu," = ",0)))

# 40%
plot(NA, xlim=c(-.93,.93), ylim=c(-.93,.93), xlab = expression(x[1]), ylab = expression(x[2]), las=1)
grid(col="grey60")
mtext("support = 40%", 3, 0.5)
rect(xleft=-.8, ybottom = -.5, xright = .8, ytop = .5)
text(0,0, expression(paste(mu," = ",delta)))
text(-.75,.75, expression(paste(mu," = ",0)))

# 2groups
# 2 x 5%
plot(NA, xlim=c(-.93,.93), ylim=c(-.93,.93), xlab = expression(x[1]), ylab = expression(x[2]), las=1)
grid(col="grey60")
mtext("support = 2 x 5%", 3, 0.5)
rect(xleft=.3, ybottom = .25, xright = .7, ytop = .75)
rect(xleft=-.7, ybottom = -.75, xright = -.3, ytop = -.25)
```

```
text(0.5,0.5, expression(paste(mu," = ",delta)))
text(-0.5,-0.5, expression(paste(mu," = ",delta)))
text(-.5,.5, expression(paste(mu," = ",0)))

# 2 x 5% (margin)
plot(NA, xlim=c(-.93,.93), ylim=c(-.93,.93), xlab = expression(x[1]), ylab = expression(x[2]), las=1)
grid(col="grey60")
mtext("support = 2 x 5% (margin)", 3, 0.5)
rect(xleft=-1, ybottom = -1, xright = -.6, ytop = -.5)
rect(xleft=.6, ybottom = .5, xright = 1, ytop = 1)
text(-.8,-.75, expression(paste(mu," = ",delta)))
text(.8,.75, expression(paste(mu," = ",delta)))
text(-.5,.5, expression(paste(mu," = ",0)))

# 2 x 10%
plot(NA, xlim=c(-.93,.93), ylim=c(-.93,.93), xlab = expression(x[1]), ylab = expression(x[2]), las=1)
grid(col="grey60")
mtext("support = 2 x 10%", 3, 0.5)
rect(xleft=.1, ybottom = .25, xright = .9, ytop = .75)
rect(xleft=-.9, ybottom = -.75, xright = -.1, ytop = -.25)
text(0.5,0.5, expression(paste(mu," = ",delta)))
text(-0.5,-0.5, expression(paste(mu," = ",delta)))
text(-.5,.5, expression(paste(mu," = ",0)))

# 2 x 20%
plot(NA, xlim=c(-.93,.93), ylim=c(-.93,.93), xlab = expression(x[1]), ylab = expression(x[2]), las=1)
grid(col="grey60")
mtext("support = 2 x 20%", 3, 0.5)
rect(xleft=.1, ybottom = 0, xright = .9, ytop = 1)
rect(xleft=-.9, ybottom = -1, xright = -.1, ytop = 0)
text(0.5,0.5, expression(paste(mu," = ",delta)))
text(-0.5,-0.5, expression(paste(mu," = ",delta)))
text(-.5,.5, expression(paste(mu," = ",0)))

dev.off()
```

Appendix D

```
# choose path for saving the results
# setwd("yourpath")

### install package PRIM (can be skipped if already done)
#install.packages("devtools")
#library(devtools)
#install_github("ao90/PRIM")

library(PRIM)
library(rpart)


s <- c(1, 2)[1]                        # number of true subgroups (stays constant for varying delta)
n <- c(250, 500, 1000)[1]                   # number of observations (stays constant for varying delta)
delta <- c(0, 1/3, 2/3, 1, 4/3, 5/3, 2, 7/3, 8/3, 3)[1] # select a signal-to-noise-ratio
# NOTE that you have to run the simulation for each delta by constant n and s to get a whole result

# initial lists for definition of the true subgroups and results of the identification methods
real5 <- real20 <- real40 <- real5m <- primopt_5 <- primopt_5m <- primopt_20 <- primopt_40 <- prim_5 <- prim_5m <-
prim_20 <- prim_40 <- tree_5 <- tree_5m <- tree_20 <- tree_40 <- pruned_tree_5 <- pruned_tree_5m <- pruned_tree_20 <-
pruned_tree_40 <- list(NULL)


########################
### start simulation ###
########################
#(CAUTION: can take a long time!)

set.seed(123) # for reproducability
for(i in 1:250){
  print(i)

  # simulate covariates
  x1 <- runif(n = n, -1, 1)
  x2 <- runif(n = n, -1, 1)
  x3 <- runif(n = n, -1, 1)
  x4 <- runif(n = n, -1, 1)
  x5 <- runif(n = n, -1, 1)
  x6 <- runif(n = n, -1, 1)

  # simulate outcome variables y for one or two true subgroups
  if (s==1){
    # support 5%
    real5[[i]] <- x1 > -.2 & x1 < .2 & x2 > -.25 & x2 < .25
    y5 <- ifelse(real5[[i]], rnorm(n,delta,1), rnorm(n,0,1))

    # support 20%
    real20[[i]] <- x1 >- .4 & x1 < .4 & x2 > -.5 & x2 < .5
    y20 <- ifelse(real20[[i]], rnorm(n,delta,1), rnorm(n,0,1))

    # support 40%
    real40[[i]] <- x1 > -.8 & x1 < .8 & x2 > -.5 & x2 < .5
    y40 <- ifelse(real40[[i]], rnorm(n,delta,1), rnorm(n,0,1))

    # support 5% margin
    real5m[[i]] <- x1 > -1 & x1 < (-.6) & x2 > -1 & x2 < (-.5)
    y5m <- ifelse(real5m[[i]], rnorm(n,delta,1), rnorm(n,0,1))
  }
  if (s==2){
    # support 2 x 5%
    real5[[i]] <- (x1>.3 & x1<.7 & x2>.25 & x2<.75) | (x1>-.7 & x1<(-.3) & x2>-.75 & x2<(-.25))
    y5 <- ifelse(real5[[i]], rnorm(n,delta,1), rnorm(n,0,1))
```

```
    # support 2 x 10%
    real20[[i]] <- (x1>.1 & x1<.9 & x2>.25 & x2<.75) | (x1>-.9 & x1<(-.1) & x2>-.75 & x2<(-.25))
    y20 <- ifelse(real20[[i]], rnorm(n,delta,1), rnorm(n,0,1))

    # support 2 x 20%
    real40[[i]] <- (x1>.1 & x1<.9 & x2>0 & x2<1) | (x1>-.9 & x1<(-.1) & x2>-1 & x2<0)
    y40 <- ifelse(real40[[i]], rnorm(n,delta,1), rnorm(n,0,1))

    # support 2 x 5% margin
    real5m[[i]] <- (x1>-1 & x1<(-.6) & x2>-1 & x2<(-.5)) | (x1>.6 & x1<1 & x2>.5 & x2<1)
    y5m <- ifelse(real5m[[i]], rnorm(n,delta,1), rnorm(n,0,1))
  }

  dat <- cbind.data.frame(y5, y20, y40, y5m, x1, x2, x3, x4, x5, x6) # create a data.frame

  # apply the three methods for the given support sizes
  if (s==1){
    primopt_5[[i]] <- PRIM(y5~x1+x2+x3+x4+x5+x6, data=dat, B = 0, peel_alpha = seq(.01, .5, .01), max_boxes = 1, beta_min =
.05, print_position = FALSE, max_steps = 20, stop_by_dec = FALSE)
    prim_5[[i]] <- PRIM(y5~x1+x2+x3+x4+x5+x6, data=dat, B = 0, peel_alpha = seq(.01, .5, .01), f_min = 2, max_boxes = 1,
beta_min = 7/n, print_position = FALSE, max_steps = 20, stop_by_dec = FALSE)
    tree_5[[i]] <- rpart(y5~x1+x2+x3+x4+x5+x6, data=dat, control = list(maxdepth = 4))
    pruned_tree_5[[i]] <- rpart(y5~x1+x2+x3+x4+x5+x6, data=dat)
    pruned_tree_5[[i]] <- prune(pruned_tree_5[[i]], cp =
pruned_tree_5[[i]]$cptable[which.min(pruned_tree_5[[i]]$cptable[,"xerror"]),"CP"])

    primopt_20[[i]] <- PRIM(y20~x1+x2+x3+x4+x5+x6, data=dat, B = 0, peel_alpha = seq(.01, .5, .01), max_boxes = 1,
beta_min = .2, print_position = FALSE, max_steps = 20, stop_by_dec = FALSE)
    prim_20[[i]] <- PRIM(y20~x1+x2+x3+x4+x5+x6, data=dat, B = 0, peel_alpha = seq(.01, .5, .01), f_min = 2, max_boxes = 1,
beta_min = 7/n, print_position = FALSE, max_steps = 20, stop_by_dec = FALSE)
    tree_20[[i]] <- rpart(y20~x1+x2+x3+x4+x5+x6, data=dat, control = list(maxdepth = 4))
    pruned_tree_20[[i]] <- rpart(y20~x1+x2+x3+x4+x5+x6, data=dat)
    pruned_tree_20[[i]] <- prune(pruned_tree_20[[i]], cp =
pruned_tree_20[[i]]$cptable[which.min(pruned_tree_20[[i]]$cptable[,"xerror"]),"CP"])

    primopt_40[[i]] <- PRIM(y40~x1+x2+x3+x4+x5+x6, data=dat, B = 0, peel_alpha = seq(.01, .5, .01), max_boxes = 1,
beta_min = .4, print_position = FALSE, max_steps = 20, stop_by_dec = FALSE)
    prim_40[[i]] <- PRIM(y40~x1+x2+x3+x4+x5+x6, data=dat, B = 0, peel_alpha = seq(.01, .5, .01), f_min = 2, max_boxes = 1,
beta_min = 7/n, print_position = FALSE, max_steps = 20, stop_by_dec = FALSE)
    tree_40[[i]] <- rpart(y40~x1+x2+x3+x4+x5+x6, data=dat, control = list(maxdepth = 4))
    pruned_tree_40[[i]] <- rpart(y40~x1+x2+x3+x4+x5+x6, data=dat)
    pruned_tree_40[[i]] <- prune(pruned_tree_40[[i]], cp =
pruned_tree_40[[i]]$cptable[which.min(pruned_tree_40[[i]]$cptable[,"xerror"]),"CP"])

    primopt_5m[[i]] <- PRIM(y5m~x1+x2+x3+x4+x5+x6, data=dat, B = 0, peel_alpha = seq(.01, .5, .01), max_boxes = 1,
beta_min = .05, print_position = FALSE, max_steps = 20, stop_by_dec = FALSE)
    prim_5m[[i]] <- PRIM(y5m~x1+x2+x3+x4+x5+x6, data=dat, B = 0, peel_alpha = seq(.01, .5, .01), f_min = 2, max_boxes = 1,
beta_min = 7/n, print_position = FALSE, max_steps = 20, stop_by_dec = FALSE)
    tree_5m[[i]] <- rpart(y5m~x1+x2+x3+x4+x5+x6, data=dat, control = list(maxdepth = 2))
    pruned_tree_5m[[i]] <- rpart(y5m~x1+x2+x3+x4+x5+x6, data=dat)
    pruned_tree_5m[[i]] <- prune(pruned_tree_5m[[i]], cp =
pruned_tree_5m[[i]]$cptable[which.min(pruned_tree_5m[[i]]$cptable[,"xerror"]),"CP"])
  }
  if (s==2){
    primopt_5[[i]] <- PRIM(y5~x1+x2+x3+x4+x5+x6, data=dat, B = 0, peel_alpha = seq(.01, .5, .01), max_boxes = 2, beta_min =
.05, print_position = FALSE, max_steps = 20, stop_by_dec = FALSE)
    prim_5[[i]] <- PRIM(y5~x1+x2+x3+x4+x5+x6, data=dat, B = 0, peel_alpha = seq(.01, .5, .01), f_min = 2, max_boxes = 2,
beta_min = 7/n, print_position = FALSE, max_steps = 20, stop_by_dec = FALSE)
    tree_5[[i]] <- rpart(y5~x1+x2+x3+x4+x5+x6, data=dat, control = list(maxdepth = 8))
    pruned_tree_5[[i]] <- rpart(y5~x1+x2+x3+x4+x5+x6, data=dat)
    pruned_tree_5[[i]] <- prune(pruned_tree_5[[i]], cp =
pruned_tree_5[[i]]$cptable[which.min(pruned_tree_5[[i]]$cptable[,"xerror"]),"CP"])
```

```
    primopt_20[[i]] <- PRIM(y20~x1+x2+x3+x4+x5+x6, data=dat, B = 0, peel_alpha = seq(.01, .5, .01), max_boxes = 2,
beta_min = .1, print_position = FALSE, max_steps = 20, stop_by_dec = FALSE)
    prim_20[[i]] <- PRIM(y20~x1+x2+x3+x4+x5+x6, data=dat, B = 0, peel_alpha = seq(.01, .5, .01), f_min = 2, max_boxes = 2,
beta_min = 7/n, print_position = FALSE, max_steps = 20, stop_by_dec = FALSE)
    tree_20[[i]] <- rpart(y20~x1+x2+x3+x4+x5+x6, data=dat, control = list(maxdepth = 8))
    pruned_tree_20[[i]] <- rpart(y20~x1+x2+x3+x4+x5+x6, data=dat)
    pruned_tree_20[[i]] <- prune(pruned_tree_20[[i]], cp =
pruned_tree_20[[i]]$cptable[which.min(pruned_tree_20[[i]]$cptable[,"xerror"]),"CP"])

    primopt_40[[i]] <- PRIM(y40~x1+x2+x3+x4+x5+x6, data=dat, B = 0, peel_alpha = seq(.01, .5, .01), max_boxes = 2,
beta_min = .2, print_position = FALSE, max_steps = 20, stop_by_dec = FALSE)
    prim_40[[i]] <- PRIM(y40~x1+x2+x3+x4+x5+x6, data=dat, B = 0, peel_alpha = seq(.01, .5, .01), f_min = 2, max_boxes = 2,
beta_min = 7/n, print_position = FALSE, max_steps = 20, stop_by_dec = FALSE)
    tree_40[[i]] <- rpart(y40~x1+x2+x3+x4+x5+x6, data=dat, control = list(maxdepth = 8))
    pruned_tree_40[[i]] <- rpart(y40~x1+x2+x3+x4+x5+x6, data=dat)
    pruned_tree_40[[i]] <- prune(pruned_tree_40[[i]], cp =
pruned_tree_40[[i]]$cptable[which.min(pruned_tree_40[[i]]$cptable[,"xerror"]),"CP"])

    primopt_5m[[i]] <- PRIM(y5m~x1+x2+x3+x4+x5+x6, data=dat, B = 0, peel_alpha = seq(.01, .5, .01), max_boxes = 2,
beta_min = .05, print_position = FALSE, max_steps = 20, stop_by_dec = FALSE)
    prim_5m[[i]] <- PRIM(y5m~x1+x2+x3+x4+x5+x6, data=dat, B = 0, peel_alpha = seq(.01, .5, .01), f_min = 2, max_boxes = 2,
beta_min = 7/n, print_position = FALSE, max_steps = 20, stop_by_dec = FALSE)
    tree_5m[[i]] <- rpart(y5m~x1+x2+x3+x4+x5+x6, data=dat, control = list(maxdepth = 4))
    pruned_tree_5m[[i]] <- rpart(y5m~x1+x2+x3+x4+x5+x6, data=dat)
    pruned_tree_5m[[i]] <- prune(pruned_tree_5m[[i]], cp =
pruned_tree_5m[[i]]$cptable[which.min(pruned_tree_5m[[i]]$cptable[,"xerror"]),"CP"])
  }

}


# save the results
save.image(paste("Sim result s", s, " n", n, " delta", round(delta, 2), "w.RData", sep = ""))
```

Appendix E

```
### CAUTION: this code runs only if all simulation results (each delta) for a constant s and n are saved

# set working directory to the path where the results are saved
# setwd("yourpath")

library(Hmisc)
library(PRIM)

##############################################
### Preparation of the simulation results ###
##############################################


# choose your number of true subgroups and observations
s <- c(1, 2)[1]
n <- c(250, 500, 1000)[1]

sens_popt5 <- sens_p5 <- sens_t5 <- sens_pt5 <- sens_popt5m <- sens_p5m <- sens_t5m <- sens_pt5m <- sens_popt20 <-
sens_p20 <- sens_t20 <- sens_pt20 <- sens_popt40 <- sens_p40 <- sens_t40 <- sens_pt40 <- NULL
spez_popt5 <- spez_p5 <- spez_t5 <- spez_pt5 <- spez_popt5m <- spez_p5m <- spez_t5m <- spez_pt5m <- spez_popt20 <-
spez_p20 <- spez_t20 <- spez_pt20 <- spez_popt40 <- spez_p40 <- spez_t40 <- spez_pt40 <- NULL
found_popt5 <- found_p5 <- found_t5 <- found_pt5 <- found_popt5m <- found_p5m <- found_t5m <- found_pt5m <-
found_popt20 <- found_p20 <- found_t20 <- found_pt20 <- found_popt40 <- found_p40 <- found_t40 <- found_pt40 <-
NULL


### takes time !!!
for(i in c(0, 0.33, 0.67, 1, 1.33, 1.67, 2, 2.33, 2.67, 3)){
 load(paste("Sim result s", s, " n", n, " delta", i, "w.RData", sep = ""))

 ### simulate test dataset
 # simulate covariates
 set.seed(1234)
 x1 <- runif(n = 10000, -1, 1)
 x2 <- runif(n = 10000, -1, 1)
 x3 <- runif(n = 10000, -1, 1)
 x4 <- runif(n = 10000, -1, 1)
 x5 <- runif(n = 10000, -1, 1)
 x6 <- runif(n = 10000, -1, 1)
 # simulate outcome variables y for one or two true subgroups
 if (s==1){
  # support 5%
  true5 <- x1 > -.2 & x1 < .2 & x2 > -.25 & x2 < .25
  y5 <- ifelse(true5, rnorm(n,delta,1), rnorm(n,0,1))
  # support 20%
  true20 <- x1 >- .4 & x1 < .4 & x2 > -.5 & x2 < .5
  y20 <- ifelse(true20, rnorm(n,delta,1), rnorm(n,0,1))
  # support 40%
  true40 <- x1 > -.8 & x1 < .8 & x2 > -.5 & x2 < .5
  y40 <- ifelse(true40, rnorm(n,delta,1), rnorm(n,0,1))
  # support 5% margin
  true5m <- x1 > -1 & x1 < (-.6) & x2 > -1 & x2 < (-.5)
  y5m <- ifelse(true5m, rnorm(n,delta,1), rnorm(n,0,1))
 }
 if (s==2){
  # support 2 x 5%
  true5 <- (x1>.3 & x1<.7 & x2>.25 & x2<.75) | (x1>-.7 & x1<(-.3) & x2>-.75 & x2<(-.25))
  y5 <- ifelse(true5, rnorm(n,delta,1), rnorm(n,0,1))
  # support 2 x 10%
  true20 <- (x1>.1 & x1<.9 & x2>.25 & x2<.75) | (x1>-.9 & x1<(-.1) & x2>-.75 & x2<(-.25))
  y20 <- ifelse(true20, rnorm(n,delta,1), rnorm(n,0,1))
  # support 2 x 20%
```

```
    true40 <- (x1>.1 & x1<.9 & x2>0 & x2<1) | (x1>-.9 & x1<(-.1) & x2>-1 & x2<0)
    y40 <- ifelse(true40, rnorm(n,delta,1), rnorm(n,0,1))
    # support 2 x 5% margin
    true5m <- (x1>-1 & x1<(-.6) & x2>-1 & x2<(-.5)) | (x1>.6 & x1<1 & x2>.5 & x2<1)
    y5m <- ifelse(true5m, rnorm(n,delta,1), rnorm(n,0,1))
  }
  test_dat <- cbind.data.frame(y5, y20, y40, y5m, x1, x2, x3, x4, x5, x6) # create a data.frame

  # save the predicted subgroups by the methods in lists
  if(s==1){
    popt5_pred <- lapply(primopt_5, function(k) {inbox(test_dat[, 4:10], fixbox_metric = k$box_metric)})
    popt5m_pred <- lapply(primopt_5m, function(k) {inbox(test_dat[, 4:10], fixbox_metric = k$box_metric)})
    popt20_pred <- lapply(primopt_20, function(k) {inbox(test_dat[, 4:10], fixbox_metric = k$box_metric)})
    popt40_pred <- lapply(primopt_40, function(k) {inbox(test_dat[, 4:10], fixbox_metric = k$box_metric)})
    p5_pred <- lapply(prim_5, function(k) {if (is.null(k$box_metric)) rep(FALSE, times=nrow(test_dat)) else inbox(test_dat[, 4:10], fixbox_metric = k$box_metric)})
    p5m_pred <- lapply(prim_5m, function(k) {if (is.null(k$box_metric)) rep(FALSE, times=nrow(test_dat)) else inbox(test_dat[, 4:10], fixbox_metric = k$box_metric)})
    p20_pred <- lapply(prim_20, function(k) {if (is.null(k$box_metric)) rep(FALSE, times=nrow(test_dat)) else inbox(test_dat[, 4:10], fixbox_metric = k$box_metric)})
    p40_pred <- lapply(prim_40, function(k) {if (is.null(k$box_metric)) rep(FALSE, times=nrow(test_dat)) else inbox(test_dat[, 4:10], fixbox_metric = k$box_metric)})
    t5_pred <- lapply(tree_5, function(k) {if(min(predict(k, test_dat[, 5:10]))==max(predict(k, test_dat[, 5:10]))) rep(FALSE, times=length(test_dat)) else {predict(k, test_dat[, 5:10])==max(predict(k, test_dat[, 5:10]))}})
    t5m_pred <- lapply(tree_5m, function(k) {if(min(predict(k, test_dat[, 5:10]))==max(predict(k, test_dat[, 5:10]))) rep(FALSE, times=length(test_dat)) else {predict(k, test_dat[, 5:10])==max(predict(k, test_dat[, 5:10]))}})
    t20_pred <- lapply(tree_20, function(k) {if(min(predict(k, test_dat[, 5:10]))==max(predict(k, test_dat[, 5:10]))) rep(FALSE, times=length(test_dat)) else {predict(k, test_dat[, 5:10])==max(predict(k, test_dat[, 5:10]))}})
    t40_pred <- lapply(tree_40, function(k) {if(min(predict(k, test_dat[, 5:10]))==max(predict(k, test_dat[, 5:10]))) rep(FALSE, times=length(test_dat)) else {predict(k, test_dat[, 5:10])==max(predict(k, test_dat[, 5:10]))}})
    pt5_pred <- lapply(pruned_tree_5, function(k) {if(min(predict(k, test_dat[, 5:10]))==max(predict(k, test_dat[, 5:10]))) rep(FALSE, times=length(test_dat)) else {predict(k, test_dat[, 5:10])==max(predict(k, test_dat[, 5:10]))}})
    pt5m_pred <- lapply(pruned_tree_5m, function(k) {if(min(predict(k, test_dat[, 5:10]))==max(predict(k, test_dat[, 5:10]))) rep(FALSE, times=length(test_dat)) else {predict(k, test_dat[, 5:10])==max(predict(k, test_dat[, 5:10]))}})
    pt20_pred <- lapply(pruned_tree_20, function(k) {if(min(predict(k, test_dat[, 5:10]))==max(predict(k, test_dat[, 5:10]))) rep(FALSE, times=length(test_dat)) else {predict(k, test_dat[, 5:10])==max(predict(k, test_dat[, 5:10]))}})
    pt40_pred <- lapply(pruned_tree_40, function(k) {if(min(predict(k, test_dat[, 5:10]))==max(predict(k, test_dat[, 5:10]))) rep(FALSE, times=length(test_dat)) else {predict(k, test_dat[, 5:10])==max(predict(k, test_dat[, 5:10]))}})
    }
  if(s==2){
    popt5_pred <- lapply(primopt_5, function(k) {inbox(test_dat[, 4:10], fixbox_metric = k$box_metric[1,]) | inbox(test_dat[, 4:10], fixbox_metric = k$box_metric[2,])})
    popt5m_pred <- lapply(primopt_5m, function(k) {inbox(test_dat[, 4:10], fixbox_metric = k$box_metric[1,]) | inbox(test_dat[, 4:10], fixbox_metric = k$box_metric[2,])})
    popt20_pred <- lapply(primopt_20, function(k) {inbox(test_dat[, 4:10], fixbox_metric = k$box_metric[1,]) | inbox(test_dat[, 4:10], fixbox_metric = k$box_metric[2,])})
    popt40_pred <- lapply(primopt_40, function(k) {inbox(test_dat[, 4:10], fixbox_metric = k$box_metric[1,]) | inbox(test_dat[, 4:10], fixbox_metric = k$box_metric[2,])})
    p5_pred <- lapply(prim_5, function(k) {if(is.null(k$box_metric)) rep(FALSE, times=nrow(test_dat)) else if(nrow(k$box_metric)==1) inbox(test_dat[, 4:10], fixbox_metric = k$box_metric[1,]) else inbox(test_dat[, 4:10], fixbox_metric = k$box_metric[1,]) | inbox(test_dat[, 4:10], fixbox_metric = k$box_metric[2,])})
    p5m_pred <- lapply(prim_5m, function(k) {if(is.null(k$box_metric)) rep(FALSE, times=nrow(test_dat)) else if(nrow(k$box_metric)==1) inbox(test_dat[, 4:10], fixbox_metric = k$box_metric[1,]) else inbox(test_dat[, 4:10], fixbox_metric = k$box_metric[1,]) | inbox(test_dat[, 4:10], fixbox_metric = k$box_metric[2,])})
    p20_pred <- lapply(prim_20, function(k) {if(is.null(k$box_metric)) rep(FALSE, times=nrow(test_dat)) else if(nrow(k$box_metric)==1) inbox(test_dat[, 4:10], fixbox_metric = k$box_metric[1,]) else inbox(test_dat[, 4:10], fixbox_metric = k$box_metric[1,]) | inbox(test_dat[, 4:10], fixbox_metric = k$box_metric[2,])})
    p40_pred <- lapply(prim_40, function(k) {if(is.null(k$box_metric)) rep(FALSE, times=nrow(test_dat)) else if(nrow(k$box_metric)==1) inbox(test_dat[, 4:10], fixbox_metric = k$box_metric[1,]) else inbox(test_dat[, 4:10], fixbox_metric = k$box_metric[1,]) | inbox(test_dat[, 4:10], fixbox_metric = k$box_metric[2,])})
    t5_pred <- lapply(tree_5, function(k) {if(min(predict(k, test_dat[, 5:10]))==max(predict(k, test_dat[, 5:10]))) rep(FALSE, times=length(test_dat)) else {predict(k, test_dat[, 5:10]) >= max(predict(k, test_dat[, 5:10])[predict(k, test_dat[, 5:10])!=max(predict(k, test_dat[, 5:10]))])}})
```

```
  t5m_pred <- lapply(tree_5m, function(k) {if(min(predict(k, test_dat[, 5:10]))==max(predict(k, test_dat[, 5:10])))
rep(FALSE, times=length(test_dat)) else {predict(k, test_dat[, 5:10]) >= max(predict(k, test_dat[, 5:10])[predict(k, test_dat[,
5:10])!=max(predict(k, test_dat[, 5:10]))])}})
  t20_pred <- lapply(tree_20, function(k) {if(min(predict(k, test_dat[, 5:10]))==max(predict(k, test_dat[, 5:10]))) rep(FALSE,
times=length(test_dat)) else {predict(k, test_dat[, 5:10]) >= max(predict(k, test_dat[, 5:10])[predict(k, test_dat[,
5:10])!=max(predict(k, test_dat[, 5:10]))])}})
  t40_pred <- lapply(tree_40, function(k) {if(min(predict(k, test_dat[, 5:10]))==max(predict(k, test_dat[, 5:10]))) rep(FALSE,
times=length(test_dat)) else {predict(k, test_dat[, 5:10]) >= max(predict(k, test_dat[, 5:10])[predict(k, test_dat[,
5:10])!=max(predict(k, test_dat[, 5:10]))])}})
  pt5_pred <- lapply(pruned_tree_5, function(k) {if(min(predict(k, test_dat[, 5:10]))==max(predict(k, test_dat[, 5:10])))
rep(FALSE, times=length(test_dat)) else {predict(k, test_dat[, 5:10]) >= max(predict(k, test_dat[, 5:10])[predict(k, test_dat[,
5:10])!=max(predict(k, test_dat[, 5:10]))])}})
  pt5m_pred <- lapply(pruned_tree_5m, function(k) {if(min(predict(k, test_dat[, 5:10]))==max(predict(k, test_dat[, 5:10])))
rep(FALSE, times=length(test_dat)) else {predict(k, test_dat[, 5:10]) >= max(predict(k, test_dat[, 5:10])[predict(k, test_dat[,
5:10])!=max(predict(k, test_dat[, 5:10]))])}})
  pt20_pred <- lapply(pruned_tree_20, function(k) {if(min(predict(k, test_dat[, 5:10]))==max(predict(k, test_dat[, 5:10])))
rep(FALSE, times=length(test_dat)) else {predict(k, test_dat[, 5:10]) >= max(predict(k, test_dat[, 5:10])[predict(k, test_dat[,
5:10])!=max(predict(k, test_dat[, 5:10]))])}})
  pt40_pred <- lapply(pruned_tree_40, function(k) {if(min(predict(k, test_dat[, 5:10]))==max(predict(k, test_dat[, 5:10])))
rep(FALSE, times=length(test_dat)) else {predict(k, test_dat[, 5:10]) >= max(predict(k, test_dat[, 5:10])[predict(k, test_dat[,
5:10])!=max(predict(k, test_dat[, 5:10]))])}})
}

 # calculate the senitivities and specificities
 sens_popt5 <- c(sens_popt5, sapply(1:length(popt5_pred), function(k) sum(true5&popt5_pred[[k]])/sum(true5)))
 sens_p5 <- c(sens_p5, sapply(1:length(p5_pred), function(k) sum(true5&p5_pred[[k]])/sum(true5)))
 sens_t5 <- c(sens_t5, sapply(1:length(t5_pred), function(k) sum(true5&t5_pred[[k]])/sum(true5)))
 sens_pt5 <- c(sens_pt5, sapply(1:length(pt5_pred), function(k) sum(true5&pt5_pred[[k]])/sum(true5)))
 spez_popt5 <- c(spez_popt5, sapply(1:length(popt5_pred), function(k)
sum(!true5&(popt5_pred[[k]]==FALSE))/sum(!true5)))
 spez_p5 <- c(spez_p5, sapply(1:length(p5_pred), function(k) sum(!true5&(p5_pred[[k]]==FALSE))/sum(!true5)))
 spez_t5 <- c(spez_t5, sapply(1:length(t5_pred), function(k) sum(!true5 & (t5_pred[[k]]==FALSE))/sum(!true5)))
 spez_pt5 <- c(spez_pt5, sapply(1:length(pt5_pred), function(k) sum(!true5 & (pt5_pred[[k]]==FALSE))/sum(!true5)))
 sens_popt5m <- c(sens_popt5m, sapply(1:length(popt5m_pred), function(k)
sum(true5m&popt5m_pred[[k]])/sum(true5m)))
 sens_p5m <- c(sens_p5m, sapply(1:length(p5m_pred), function(k) sum(true5m&p5m_pred[[k]])/sum(true5m)))
 sens_t5m <- c(sens_t5m, sapply(1:length(t5m_pred), function(k) sum(true5m&t5m_pred[[k]])/sum(true5m)))
 sens_pt5m <- c(sens_pt5m, sapply(1:length(pt5m_pred), function(k) sum(true5m&pt5m_pred[[k]])/sum(true5m)))
 spez_popt5m <- c(spez_popt5m, sapply(1:length(popt5m_pred), function(k)
sum(!true5m&(popt5m_pred[[k]]==FALSE))/sum(!true5m)))
 spez_p5m <- c(spez_p5m, sapply(1:length(p5m_pred), function(k) sum(!true5m&(p5m_pred[[k]]==FALSE))/sum(!true5m)))
 spez_t5m <- c(spez_t5m, sapply(1:length(t5m_pred), function(k) sum(!true5m & (t5m_pred[[k]]==FALSE))/sum(!true5m)))
 spez_pt5m <- c(spez_pt5m, sapply(1:length(pt5m_pred), function(k) sum(!true5m &
(pt5m_pred[[k]]==FALSE))/sum(!true5m)))
 sens_popt20 <- c(sens_popt20, sapply(1:length(popt20_pred), function(k) sum(true20&popt20_pred[[k]])/sum(true20)))
 sens_p20 <- c(sens_p20, sapply(1:length(p20_pred), function(k) sum(true20&p20_pred[[k]])/sum(true20)))
 sens_t20 <- c(sens_t20, sapply(1:length(t20_pred), function(k) sum(true20&t20_pred[[k]])/sum(true20)))
 sens_pt20 <- c(sens_pt20, sapply(1:length(pt20_pred), function(k) sum(true20&pt20_pred[[k]])/sum(true20)))
 spez_popt20 <- c(spez_popt20, sapply(1:length(popt20_pred), function(k)
sum(!true20&(popt20_pred[[k]]==FALSE))/sum(!true20)))
 spez_p20 <- c(spez_p20, sapply(1:length(p20_pred), function(k) sum(!true20&(p20_pred[[k]]==FALSE))/sum(!true20)))
 spez_t20 <- c(spez_t20, sapply(1:length(t20_pred), function(k) sum(!true20 & (t20_pred[[k]]==FALSE))/sum(!true20)))
 spez_pt20 <- c(spez_pt20, sapply(1:length(pt20_pred), function(k) sum(!true20 & (pt20_pred[[k]]==FALSE))/sum(!true20)))
 sens_popt40 <- c(sens_popt40, sapply(1:length(popt40_pred), function(k) sum(true40&popt40_pred[[k]])/sum(true40)))
 sens_p40 <- c(sens_p40, sapply(1:length(p40_pred), function(k) sum(true40&p40_pred[[k]])/sum(true40)))
 sens_t40 <- c(sens_t40, sapply(1:length(t40_pred), function(k) sum(true40&t40_pred[[k]])/sum(true40)))
 sens_pt40 <- c(sens_pt40, sapply(1:length(pt40_pred), function(k) sum(true40&pt40_pred[[k]])/sum(true40)))
 spez_popt40 <- c(spez_popt40, sapply(1:length(popt40_pred), function(k)
sum(!true40&(popt40_pred[[k]]==FALSE))/sum(!true40)))
 spez_p40 <- c(spez_p40, sapply(1:length(p40_pred), function(k) sum(!true40&(p40_pred[[k]]==FALSE))/sum(!true40)))
 spez_t40 <- c(spez_t40, sapply(1:length(t40_pred), function(k) sum(!true40 & (t40_pred[[k]]==FALSE))/sum(!true40)))
 spez_pt40 <- c(spez_pt40, sapply(1:length(pt40_pred), function(k) sum(!true40 & (pt40_pred[[k]]==FALSE))/sum(!true40)))

 # create vectors that specify if a method found a subgroup for each run
 found_popt5 <- c(found_popt5, sapply(primopt_5, function(k) sum(k$subsets[[1]])>0))
```

```
  found_p5 <- c(found_p5, sapply(prim_5, function(k) sum(k$subsets[[1]])>0))
  found_t5 <- c(found_t5, sapply(tree_5, function(k) mean(predict(k)==max(predict(k)))!=1))
  found_pt5 <- c(found_pt5, sapply(pruned_tree_5, function(k) mean(predict(k)==max(predict(k)))!=1))
  found_popt5m <- c(found_popt5m, sapply(primopt_5m, function(k) sum(k$subsets[[1]])>0))
  found_p5m <- c(found_p5m, sapply(prim_5m, function(k) sum(k$subsets[[1]])>0))
  found_t5m <- c(found_t5m, sapply(tree_5m, function(k) mean(predict(k)==max(predict(k)))!=1))
  found_pt5m <- c(found_pt5m, sapply(pruned_tree_5m, function(k) mean(predict(k)==max(predict(k)))!=1))
  found_popt20 <- c(found_popt20, sapply(primopt_20, function(k) sum(k$subsets[[1]])>0))
  found_p20 <- c(found_p20, sapply(prim_20, function(k) sum(k$subsets[[1]])>0))
  found_t20 <- c(found_t20, sapply(tree_20, function(k) mean(predict(k)==max(predict(k)))!=1))
  found_pt20 <- c(found_pt20, sapply(pruned_tree_20, function(k) mean(predict(k)==max(predict(k)))!=1))
  found_popt40 <- c(found_popt40, sapply(primopt_40, function(k) sum(k$subsets[[1]])>0))
  found_p40 <- c(found_p40, sapply(prim_40, function(k) sum(k$subsets[[1]])>0))
  found_t40 <- c(found_t40, sapply(tree_40, function(k) mean(predict(k)==max(predict(k)))!=1))
  found_pt40 <- c(found_pt40, sapply(pruned_tree_40, function(k) mean(predict(k)==max(predict(k)))!=1))
}

# corresponding delta vector defining which element of the results (e.g. sens_popt5) belongs to which delta value
d <- c(0, 1/3, 2/3, 1, 1+1/3, 1+2/3, 2, 2+1/3, 2+2/3, 3)
delta <- rep(d, each=250)

# delete objects that are not used anymore
remove(real5, real20, real40, primopt_5, primopt_20, primopt_40, prim_5, prim_20, prim_40, tree_5, tree_20, tree_40)


### calculate the Youden's J statistics from sensitivities and specificities:
y_popt5<-(sens_popt5+spez_popt5)-1
y_p5<-(sens_p5+spez_p5)-1
y_t5<-(sens_t5+spez_t5)-1
y_pt5<-(sens_pt5+spez_pt5)-1
y_popt5m<-(sens_popt5m+spez_popt5m)-1
y_p5m<-(sens_p5m+spez_p5m)-1
y_t5m<-(sens_t5m+spez_t5m)-1
y_pt5m<-(sens_pt5m+spez_pt5m)-1
y_popt20<-(sens_popt20+spez_popt20)-1
y_p20<-(sens_p20+spez_p20)-1
y_t20<-(sens_t20+spez_t20)-1
y_pt20<-(sens_pt20+spez_pt20)-1
y_popt40<-(sens_popt40+spez_popt40)-1
y_p40<-(sens_p40+spez_p40)-1
y_t40<-(sens_t40+spez_t40)-1
y_pt40<-(sens_pt40+spez_pt40)-1


### calculate the 5%- 50%- and 95%-confidence intervals for sensitivities, specificities and Youden's J statistics
# support = 5%
q_popt_spez5 <- sapply(d, function(k) quantile(spez_popt5[delta==k], probs = c(.25,.5,.75)))
q_p_spez5 <- sapply(d, function(k) quantile(spez_p5[delta==k], probs = c(.25,.5,.75)))
q_t_spez5 <- sapply(d, function(k) quantile(spez_t5[delta==k], probs = c(.25,.5,.75)))
q_pt_spez5 <- sapply(d, function(k) quantile(spez_pt5[delta==k], probs = c(.25,.5,.75)))
q_popt_sens5 <- sapply(d, function(k) quantile(sens_popt5[delta==k], probs = c(.25,.5,.75)))
q_p_sens5 <- sapply(d, function(k) quantile(sens_p5[delta==k], probs = c(.25,.5,.75)))
q_t_sens5 <- sapply(d, function(k) quantile(sens_t5[delta==k], probs = c(.25,.5,.75)))
q_pt_sens5 <- sapply(d, function(k) quantile(sens_pt5[delta==k], probs = c(.25,.5,.75)))
q_popt_y5 <- sapply(d, function(k) quantile(y_popt5[delta==k], probs = c(.25,.5,.75)))
q_p_y5 <- sapply(d, function(k) quantile(y_p5[delta==k], probs = c(.25,.5,.75)))
q_t_y5 <- sapply(d, function(k) quantile(y_t5[delta==k], probs = c(.25,.5,.75)))
q_pt_y5 <- sapply(d, function(k) quantile(y_pt5[delta==k], probs = c(.25,.5,.75)))
# support = 5% (margin)
q_popt_spez5m <- sapply(d, function(k) quantile(spez_popt5m[delta==k], probs = c(.25,.5,.75)))
q_p_spez5m <- sapply(d, function(k) quantile(spez_p5m[delta==k], probs = c(.25,.5,.75)))
q_t_spez5m <- sapply(d, function(k) quantile(spez_t5m[delta==k], probs = c(.25,.5,.75)))
q_pt_spez5m <- sapply(d, function(k) quantile(spez_pt5m[delta==k], probs = c(.25,.5,.75)))
q_popt_sens5m <- sapply(d, function(k) quantile(sens_popt5m[delta==k], probs = c(.25,.5,.75)))
q_p_sens5m <- sapply(d, function(k) quantile(sens_p5m[delta==k], probs = c(.25,.5,.75)))
```

```
q_t_sens5m <- sapply(d, function(k) quantile(sens_t5m[delta==k], probs = c(.25,.5,.75)))
q_pt_sens5m <- sapply(d, function(k) quantile(sens_pt5m[delta==k], probs = c(.25,.5,.75)))
q_popt_y5m <- sapply(d, function(k) quantile(y_popt5m[delta==k], probs = c(.25,.5,.75)))
q_p_y5m <- sapply(d, function(k) quantile(y_p5m[delta==k], probs = c(.25,.5,.75)))
q_t_y5m <- sapply(d, function(k) quantile(y_t5m[delta==k], probs = c(.25,.5,.75)))
q_pt_y5m <- sapply(d, function(k) quantile(y_pt5m[delta==k], probs = c(.25,.5,.75)))
# support = 20%
q_popt_spez20 <- sapply(d, function(k) quantile(spez_popt20[delta==k], probs = c(.25,.5,.75)))
q_p_spez20 <- sapply(d, function(k) quantile(spez_p20[delta==k], probs = c(.25,.5,.75)))
q_t_spez20 <- sapply(d, function(k) quantile(spez_t20[delta==k], probs = c(.25,.5,.75)))
q_pt_spez20 <- sapply(d, function(k) quantile(spez_pt20[delta==k], probs = c(.25,.5,.75)))
q_popt_sens20 <- sapply(d, function(k) quantile(sens_popt20[delta==k], probs = c(.25,.5,.75)))
q_p_sens20 <- sapply(d, function(k) quantile(sens_p20[delta==k], probs = c(.25,.5,.75)))
q_t_sens20 <- sapply(d, function(k) quantile(sens_t20[delta==k], probs = c(.25,.5,.75)))
q_pt_sens20 <- sapply(d, function(k) quantile(sens_pt20[delta==k], probs = c(.25,.5,.75)))
q_popt_y20 <- sapply(d, function(k) quantile(y_popt20[delta==k], probs = c(.25,.5,.75)))
q_p_y20 <- sapply(d, function(k) quantile(y_p20[delta==k], probs = c(.25,.5,.75)))
q_t_y20 <- sapply(d, function(k) quantile(y_t20[delta==k], probs = c(.25,.5,.75)))
q_pt_y20 <- sapply(d, function(k) quantile(y_pt20[delta==k], probs = c(.25,.5,.75)))
# support = 40%
q_popt_spez40 <- sapply(d, function(k) quantile(spez_popt40[delta==k], probs = c(.25,.5,.75)))
q_p_spez40 <- sapply(d, function(k) quantile(spez_p40[delta==k], probs = c(.25,.5,.75)))
q_t_spez40 <- sapply(d, function(k) quantile(spez_t40[delta==k], probs = c(.25,.5,.75)))
q_pt_spez40 <- sapply(d, function(k) quantile(spez_pt40[delta==k], probs = c(.25,.5,.75)))
q_popt_sens40 <- sapply(d, function(k) quantile(sens_popt40[delta==k], probs = c(.25,.5,.75)))
q_p_sens40 <- sapply(d, function(k) quantile(sens_p40[delta==k], probs = c(.25,.5,.75)))
q_t_sens40 <- sapply(d, function(k) quantile(sens_t40[delta==k], probs = c(.25,.5,.75)))
q_pt_sens40 <- sapply(d, function(k) quantile(sens_pt40[delta==k], probs = c(.25,.5,.75)))
q_popt_y40 <- sapply(d, function(k) quantile(y_popt40[delta==k], probs = c(.25,.5,.75)))
q_p_y40 <- sapply(d, function(k) quantile(y_p40[delta==k], probs = c(.25,.5,.75)))
q_t_y40 <- sapply(d, function(k) quantile(y_t40[delta==k], probs = c(.25,.5,.75)))
q_pt_y40 <- sapply(d, function(k) quantile(y_pt40[delta==k], probs = c(.25,.5,.75)))


##########################################################
### graphical illustration of the simulation results ###
##########################################################

at1 <- d[-1]-.06
at2 <- d[-1]-.02
at3 <- d[-1]+.02
at4 <- d[-1]+.06

postscript(paste("result_s", s, "_n", n, ".eps", sep = ""), width=10, height=7.5, horizontal = F, paper = "special")
par(mfrow=c(3,4), mar=c(0.5,0.5,0,0), oma=c(4.5,4.5,4,2))

plot(NULL,ylim=c(0,1), xlim=c(0,3), ylab="", xlab=expression(delta), main="", las=1, xaxt="n")
if(s==1) mtext("support = 5%", line = 2) else mtext("support = 2 x 5%", line = 2)
mtext("sensitivity", line = 3, side=2)
errbar(x = at1, y = q_p_sens5[2,-1], yminus = q_p_sens5[1,-1], yplus = q_p_sens5[3,-1], add = T, errbar.col = "green2",
col="green2",  cap = .05, lty=2)
errbar(x = at4, y = q_t_sens5[2,-1], yminus = q_t_sens5[1,-1], yplus = q_t_sens5[3,-1], errbar.col = 1, col=1, pch=15, add = T,
cap = .05, lty=2)
errbar(x = at3, y = q_pt_sens5[2,-1], yminus = q_pt_sens5[1,-1], yplus = q_pt_sens5[3,-1], errbar.col = 2, col=2, pch=18, add
= T,  cap = .05, lty=2)
errbar(x = at2, y = q_popt_sens5[2,-1], yminus = q_popt_sens5[1,-1], yplus = q_popt_sens5[3,-1],errbar.col =
adjustcolor("blue", offset = c(0,.25,.25,.25)), col=adjustcolor("blue", offset = c(0,.25,.25,.25)), pch=17, add = T, cap = .05,
lty=2)
lines(at1, q_p_sens5[2,-1], col="green2", ylim=c(0,1), type="b", las=1, xlab=expression(delta), oma=c(1,4,3,0), lwd=2,
cex=1.5, pch=16)
lines(at4, q_t_sens5[2,-1], col=1, pch=15, type="b",lwd=2, cex=1.5)
lines(at3, q_pt_sens5[2,-1], col=2, pch=18, type="b",lwd=2, cex=1.5)
lines(at2, q_popt_sens5[2,-1], col=adjustcolor("blue", offset = c(0,.25,.25,.25)), pch=17, type="b", lwd=2, cex=1.5)
axis(1, at=seq(0,3,.5), labels = rep("", 7))
```

```
plot(NULL,ylim=c(0,1), xlim=c(0,3), ylab="", xlab=expression(delta), main="", las=1, xaxt="n", yaxt="n")
if(s==1) mtext("support = 5% (margin)", line = 2) else mtext("support = 2 x 5% (margin)", line = 2)
errbar(x = at1, y = q_p_sens5m[2,-1], yminus = q_p_sens5m[1,-1], yplus = q_p_sens5m[3,-1], add = T, errbar.col = "green2",
col="green2",  cap = .05, lty=2)
errbar(x = at4, y = q_t_sens5m[2,-1], yminus = q_t_sens5m[1,-1], yplus = q_t_sens5m[3,-1], errbar.col = 1, col=1, pch=15,
add = T,  cap = .05, lty=2)
errbar(x = at3, y = q_pt_sens5m[2,-1], yminus = q_pt_sens5m[1,-1], yplus = q_pt_sens5m[3,-1], errbar.col = 2, col=2,
pch=18, add = T,  cap = .05, lty=2)
errbar(x = at2, y = q_popt_sens5m[2,-1], yminus = q_popt_sens5m[1,-1], yplus = q_popt_sens5m[3,-1],errbar.col =
adjustcolor("blue", offset = c(0,.25,.25,.25)), col=adjustcolor("blue", offset = c(0,.25,.25,.25)), pch=17, add = T,  cap = .05,
lty=2)
lines(at1, q_p_sens5m[2,-1], col="green2", ylim=c(0,1), type="b", las=1, xlab=expression(delta), oma=c(1,4,3,0), lwd=2,
cex=1.5, pch=16)
lines(at4, q_t_sens5m[2,-1], col=1, pch=15, type="b",lwd=2, cex=1.5)
lines(at3, q_pt_sens5m[2,-1], col=2, pch=18, type="b",lwd=2, cex=1.5)
lines(at2, q_popt_sens5m[2,-1], col=adjustcolor("blue", offset = c(0,.25,.25,.25)), pch=17, type="b", lwd=2, cex=1.5)
axis(1, at=seq(0,3,.5), labels = rep("", 7))
axis(2, at=seq(0,1,.2), labels = rep("", 6))

plot(NULL,ylim=c(0,1), xlim=c(0,3), ylab="", xlab=expression(delta), main="", las=1, xaxt="n", yaxt="n")
if(s==1) mtext("support = 20%", line = 2) else mtext("support = 2 x 10%", line = 2)
errbar(x = at1, y = q_p_sens20[2,-1], yminus = q_p_sens20[1,-1], yplus = q_p_sens20[3,-1], add = T, errbar.col = "green2",
col="green2",  cap = .05, lty=2)
errbar(x = at4, y = q_t_sens20[2,-1], yminus = q_t_sens20[1,-1], yplus = q_t_sens20[3,-1], errbar.col = 1, col=1, pch=15, add
= T,  cap = .05, lty=2)
errbar(x = at3, y = q_pt_sens20[2,-1], yminus = q_pt_sens20[1,-1], yplus = q_pt_sens20[3,-1], errbar.col = 2, col=2, pch=18,
add = T,  cap = .05, lty=2)
errbar(x = at2, y = q_popt_sens20[2,-1], yminus = q_popt_sens20[1,-1], yplus = q_popt_sens20[3,-1],errbar.col =
adjustcolor("blue", offset = c(0,.25,.25,.25)), col=adjustcolor("blue", offset = c(0,.25,.25,.25)), pch=17, add = T,  cap = .05,
lty=2)
lines(at1, q_p_sens20[2,-1], col="green2", ylim=c(0,1), type="b", las=1, ylab="", xlab=expression(delta), oma=c(1,4,3,0),
lwd=2, cex=1.5, pch=16)
lines(at4, q_t_sens20[2,-1], col=1, pch=15, type="b", lwd=2, cex=1.5)
lines(at3, q_pt_sens20[2,-1], col=2, pch=18, type="b",lwd=2, cex=1.5)
lines(at2, q_popt_sens20[2,-1], col=adjustcolor("blue", offset = c(0,.25,.25,.25)), pch=17, type="b", lwd=2, cex=1.5)
axis(1, at=seq(0,3,.5), labels = rep("", 7))
axis(2, at=seq(0,1,.2), labels = rep("", 6))

plot(NULL,ylim=c(0,1), xlim=c(0,3), ylab="", xlab=expression(delta), main="", las=1, xaxt="n", yaxt="n")
if(s==1) mtext("support = 40%", line = 2) else mtext("support = 2 x 20%", line = 2)
errbar(x = at1, y = q_p_sens40[2,-1], yminus = q_p_sens40[1,-1], yplus = q_p_sens40[3,-1], add = T, errbar.col = "green2",
col="green2",  cap = .05, lty=2)
errbar(x = at4, y = q_t_sens40[2,-1], yminus = q_t_sens40[1,-1], yplus = q_t_sens40[3,-1], errbar.col = 1, col=1, pch=15, add
= T,  cap = .05, lty=2)
errbar(x = at3, y = q_pt_sens40[2,-1], yminus = q_pt_sens40[1,-1], yplus = q_pt_sens40[3,-1], errbar.col = 2, col=2, pch=18,
add = T,  cap = .05, lty=2)
errbar(x = at2, y = q_popt_sens40[2,-1], yminus = q_popt_sens40[1,-1], yplus = q_popt_sens40[3,-1],errbar.col =
adjustcolor("blue", offset = c(0,.25,.25,.25)), col=adjustcolor("blue", offset = c(0,.25,.25,.25)), pch=17, add = T,  cap = .05,
lty=2)
lines(at1, q_p_sens40[2,-1], col="green2", ylim=c(0,1), type="b", las=1, xlab=expression(delta), oma=c(1,4,3,0), lwd=2,
cex=1.5, pch=16)
lines(at4, q_t_sens40[2,-1], col=1, pch=15, type="b", lwd=2, cex=1.5)
lines(at3, q_pt_sens40[2,-1], col=2, pch=18, type="b",lwd=2, cex=1.5)
lines(at2, q_popt_sens40[2,-1], col=adjustcolor("blue", offset = c(0,.25,.25,.25)), pch=17, type="b", lwd=2, cex=1.5)
axis(1, at=seq(0,3,.5), labels = rep("", 7))
axis(2, at=seq(0,1,.2), labels = rep("", 6))

plot(NULL,ylim=c(0,1), xlim=c(0,3), ylab="", xlab=expression(delta), main="", las=1, xaxt="n")
mtext("specificity", line = 3, side=2)
errbar(x = at1, y = q_p_spez5[2,-1], yminus = q_p_spez5[1,-1], yplus = q_p_spez5[3,-1], add = T, errbar.col = "green2",
col="green2",  cap = .05, lty=2)
errbar(x = at4, y = q_t_spez5[2,-1], yminus = q_t_spez5[1,-1], yplus = q_t_spez5[3,-1], errbar.col = 1, col=1, pch=15, add = T,
cap = .05, lty=2)
errbar(x = at3, y = q_pt_spez5[2,-1], yminus = q_pt_spez5[1,-1], yplus = q_pt_spez5[3,-1], errbar.col = 2, col=2, pch=18, add
= T,  cap = .05, lty=2)
```

```
errbar(x = at2, y = q_popt_spez5[2,-1], yminus = q_popt_spez5[1,-1], yplus = q_popt_spez5[3,-1],errbar.col =
adjustcolor("blue", offset = c(0,.25,.25,.25)), col=adjustcolor("blue", offset = c(0,.25,.25,.25)), pch=17, add = T,  cap = .05,
lty=2)
lines(at1, q_p_spez5[2,-1],  col="green2", type="b", lwd=2, cex=1.5, pch=16)
lines(at4, q_t_spez5[2,-1], col=1, pch=15, type="b", lwd=2, cex=1.5)
lines(at3, q_pt_spez5[2,-1], col=2, pch=18, type="b",lwd=2, cex=1.5)
lines(at2, q_popt_spez5[2,-1], col=adjustcolor("blue", offset = c(0,.25,.25,.25)), pch=17, type="b", lwd=2, cex=1.5)
axis(1, at=seq(0,3,.5), labels = rep("", 7))
legend("center", legend = c(expression(paste("PRIM (",f[min], "= 2)")), expression(paste("PRIM (opt. ",beta, ")", sep = "")),
"CART (pruned)", "CART (maxdepth)"), col=c("green2", adjustcolor("blue", offset = c(0,.25,.25,.25)),2,1), pch=c(16,17,18,15),
bty = "n", pt.cex = 1.5)


plot(NULL,ylim=c(0,1), xlim=c(0,3), ylab="", xlab=expression(delta), main="", las=1, xaxt="n", yaxt="n")
errbar(x = at1, y = q_p_spez5m[2,-1], yminus = q_p_spez5m[1,-1], yplus = q_p_spez5m[3,-1], add = T, errbar.col = "green2",
col="green2",  cap = .05, lty=2)
errbar(x = at4, y = q_t_spez5m[2,-1], yminus = q_t_spez5m[1,-1], yplus = q_t_spez5m[3,-1], errbar.col = 1, col=1, pch=15,
add = T,  cap = .05, lty=2)
errbar(x = at3, y = q_pt_spez5m[2,-1], yminus = q_pt_spez5m[1,-1], yplus = q_pt_spez5m[3,-1], errbar.col = 2, col=2,
pch=18, add = T,  cap = .05, lty=2)
errbar(x = at2, y = q_popt_spez5m[2,-1], yminus = q_popt_spez5m[1,-1], yplus = q_popt_spez5m[3,-1],errbar.col =
adjustcolor("blue", offset = c(0,.25,.25,.25)), col=adjustcolor("blue", offset = c(0,.25,.25,.25)), pch=17, add = T,  cap = .05,
lty=2)
lines(at1, q_p_spez5m[2,-1],  col="green2", type="b", lwd=2, cex=1.5, pch=16)
lines(at4, q_t_spez5m[2,-1], col=1, pch=15, type="b", lwd=2, cex=1.5)
lines(at3, q_pt_spez5m[2,-1], col=2, pch=18, type="b",lwd=2, cex=1.5)
lines(at2, q_popt_spez5m[2,-1], col=adjustcolor("blue", offset = c(0,.25,.25,.25)), pch=17, type="b", lwd=2, cex=1.5)
axis(1, at=seq(0,3,.5), labels = rep("", 7))
axis(2, at=seq(0,1,.2), labels = rep("", 6))

plot(NULL,ylim=c(0,1), xlim=c(0,3), ylab="", xlab=expression(delta), main="", las=1, xaxt="n", yaxt="n")
errbar(x = at1, y = q_p_spez20[2,-1], yminus = q_p_spez20[1,-1], yplus = q_p_spez20[3,-1], add = T, errbar.col = "green2",
col="green2",  cap = .05, lty=2)
errbar(x = at4, y = q_t_spez20[2,-1], yminus = q_t_spez20[1,-1], yplus = q_t_spez20[3,-1], errbar.col = 1, col=1, pch=15, add
= T,  cap = .05, lty=2)
errbar(x = at3, y = q_pt_spez20[2,-1], yminus = q_pt_spez20[1,-1], yplus = q_pt_spez20[3,-1], errbar.col = 2, col=2, pch=18,
add = T,  cap = .05, lty=2)
errbar(x = at2, y = q_popt_spez20[2,-1], yminus = q_popt_spez20[1,-1], yplus = q_popt_spez20[3,-1],errbar.col =
adjustcolor("blue", offset = c(0,.25,.25,.25)), col=adjustcolor("blue", offset = c(0,.25,.25,.25)), pch=17, add = T,  cap = .05,
lty=2)
lines(at1, q_p_spez20[2,-1],  col="green2", type="b", lwd=2, cex=1.5, pch=16)
lines(at4, q_t_spez20[2,-1], col=1, pch=15, type="b", lwd=2, cex=1.5)
lines(at3, q_pt_spez20[2,-1], col=2, pch=18, type="b",lwd=2, cex=1.5)
lines(at2, q_popt_spez20[2,-1], col=adjustcolor("blue", offset = c(0,.25,.25,.25)), pch=17, type="b", lwd=2, cex=1.5)
axis(1, at=seq(0,3,.5), labels = rep("", 7))
axis(2, at=seq(0,1,.2), labels = rep("", 6))

plot(NULL,ylim=c(0,1), xlim=c(0,3), ylab="", xlab=expression(delta), main="", las=1, xaxt="n", yaxt="n")
errbar(x = at1, y = q_p_spez40[2,-1], yminus = q_p_spez40[1,-1], yplus = q_p_spez40[3,-1], add = T, errbar.col = "green2",
col="green2",  cap = .05, lty=2)
errbar(x = at4, y = q_t_spez40[2,-1], yminus = q_t_spez40[1,-1], yplus = q_t_spez40[3,-1], errbar.col = 1, col=1, pch=15, add
= T,  cap = .05, lty=2)
errbar(x = at3, y = q_pt_spez40[2,-1], yminus = q_pt_spez40[1,-1], yplus = q_pt_spez40[3,-1], errbar.col = 2, col=2, pch=18,
add = T,  cap = .05, lty=2)
errbar(x = at2, y = q_popt_spez40[2,-1], yminus = q_popt_spez40[1,-1], yplus = q_popt_spez40[3,-1],errbar.col =
adjustcolor("blue", offset = c(0,.25,.25,.25)), col=adjustcolor("blue", offset = c(0,.25,.25,.25)), pch=17, add = T,  cap = .05,
lty=2)
lines(at1, q_p_spez40[2,-1],  col="green2", type="b", lwd=2, cex=1.5, pch=16)
lines(at4, q_t_spez40[2,-1], col=1, pch=15, type="b", lwd=2, cex=1.5)
lines(at3, q_pt_spez40[2,-1], col=2, pch=18, type="b",lwd=2, cex=1.5)
lines(at2, q_popt_spez40[2,-1], col=adjustcolor("blue", offset = c(0,.25,.25,.25)), pch=17, type="b", lwd=2, cex=1.5)
axis(1, at=seq(0,3,.5), labels = rep("", 7))
axis(2, at=seq(0,1,.2), labels = rep("", 6))

plot(NULL, xlim=c(0,3), ylim=c(-.1,1), las=1, xlab=expression(delta), ylab="")
```

```
mtext(expression(delta), 1, 3)
mtext("Youden's J", line = 3, side=2)
errbar(x = at1, y = q_p_y5[2,-1], yminus = q_p_y5[1,-1], yplus = q_p_y5[3,-1], add = T, errbar.col = "green2", col="green2",
cap = .05, lty=2)
errbar(x = at4, y = q_t_y5[2,-1], yminus = q_t_y5[1,-1], yplus = q_t_y5[3,-1], pch=15, add = T, col=1, errbar.col=1,  cap = .05,
lty=2)
errbar(x = at3, y = q_pt_y5[2,-1], yminus = q_pt_y5[1,-1], yplus = q_pt_y5[3,-1], pch=18, add = T, col=2, errbar.col=2,  cap =
.05, lty=2)
errbar(x = at2, y = q_popt_y5[2,-1], yminus = q_popt_y5[1,-1], yplus = q_popt_y5[3,-1],pch = 17, add = T,
col=adjustcolor("blue", offset = c(0,.25,.25,.25)), errbar.col=adjustcolor("blue", offset = c(0,.25,.25,.25)),  cap = .05, lty=2)
lines(at1, q_p_y5[2,-1], type="b", col="green2", lwd=2, cex=1.5, pch=16)
lines(at4, q_t_y5[2,-1],   pch=15, type="b", col=1, lwd=2, cex=1.5)
lines(at3, q_pt_y5[2,-1],   pch=18, type="b", col=2, lwd=2, cex=1.5)
lines(at2, q_popt_y5[2,-1], pch=17, type="b", col=adjustcolor("blue", offset = c(0,.25,.25,.25)), lwd=2, cex=1.5)

plot(NULL, xlim=c(0,3), ylim=c(-.1,1), las=1, xlab=expression(delta), ylab="", yaxt="n")
mtext(expression(delta), 1, 3)
errbar(x = at1, y = q_p_y5m[2,-1], yminus = q_p_y5m[1,-1], yplus = q_p_y5m[3,-1], add = T, errbar.col = "green2",
col="green2",  cap = .05, lty=2)
errbar(x = at4, y = q_t_y5m[2,-1], yminus = q_t_y5m[1,-1], yplus = q_t_y5m[3,-1], pch=15, add = T, col=1, errbar.col=1,  cap
= .05, lty=2)
errbar(x = at3, y = q_pt_y5m[2,-1], yminus = q_pt_y5m[1,-1], yplus = q_pt_y5m[3,-1], pch=18, add = T, col=2, errbar.col=2,
cap = .05, lty=2)
errbar(x = at2, y = q_popt_y5m[2,-1], yminus = q_popt_y5m[1,-1], yplus = q_popt_y5m[3,-1],pch = 17, add = T,
col=adjustcolor("blue", offset = c(0,.25,.25,.25)), errbar.col=adjustcolor("blue", offset = c(0,.25,.25,.25)),  cap = .05, lty=2)
lines(at1, q_p_y5m[2,-1], type="b", col="green2", lwd=2, cex=1.5, pch=16)
lines(at4, q_t_y5m[2,-1],   pch=15, type="b", col=1, lwd=2, cex=1.5)
lines(at3, q_pt_y5m[2,-1],   pch=18, type="b", col=2, lwd=2, cex=1.5)
lines(at2, q_popt_y5m[2,-1], pch=17, type="b", col=adjustcolor("blue", offset = c(0,.25,.25,.25)), lwd=2, cex=1.5)
axis(2, at=seq(0,1,.2), labels = rep("", 6))

plot(NULL, xlim=c(0,3), ylim=c(-.1,1), las=1, xlab=expression(delta), ylab="", yaxt="n")
mtext(expression(delta), 1, 3)
errbar(x = at1, y = q_p_y20[2,-1], yminus = q_p_y20[1,-1], yplus = q_p_y20[3,-1], add = T, errbar.col = "green2",
col="green2",  cap = .05, lty=2)
errbar(x = at4, y = q_t_y20[2,-1], yminus = q_t_y20[1,-1], yplus = q_t_y20[3,-1], pch=15, add = T, col=1, errbar.col=1,  cap =
.05, lty=2)
errbar(x = at3, y = q_pt_y20[2,-1], yminus = q_pt_y20[1,-1], yplus = q_pt_y20[3,-1], pch=18, add = T, col=2, errbar.col=2,
cap = .05, lty=2)
errbar(x = at2, y = q_popt_y20[2,-1], yminus = q_popt_y20[1,-1], yplus = q_popt_y20[3,-1],pch = 17, add = T,
col=adjustcolor("blue", offset = c(0,.25,.25,.25)), errbar.col=adjustcolor("blue", offset = c(0,.25,.25,.25)),  cap = .05, lty=2)
lines(at1, q_p_y20[2,-1], type="b", col="green2", lwd=2, cex=1.5, pch=16)
lines(at4, q_t_y20[2,-1],   pch=15, type="b", col=1, lwd=2, cex=1.5)
lines(at3, q_pt_y20[2,-1],   pch=18, type="b", col=2, lwd=2, cex=1.5)
lines(at2, q_popt_y20[2,-1], pch=17, type="b", col=adjustcolor("blue", offset = c(0,.25,.25,.25)), lwd=2, cex=1.5)
axis(2, at=seq(0,1,.2), labels = rep("", 6))

plot(NULL, xlim=c(0,3), ylim=c(-.1,1), las=1, xlab=expression(delta), ylab="", yaxt="n")
mtext(expression(delta), 1, 3)
errbar(x = at1, y = q_p_y40[2,-1], yminus = q_p_y40[1,-1], yplus = q_p_y40[3,-1], add = T, errbar.col = "green2",
col="green2",  cap = .05, lty=2)
errbar(x = at4, y = q_t_y40[2,-1], yminus = q_t_y40[1,-1], yplus = q_t_y40[3,-1], pch=15, add = T, col=1, errbar.col=1,  cap =
.05, lty=2)
errbar(x = at3, y = q_pt_y40[2,-1], yminus = q_pt_y40[1,-1], yplus = q_pt_y40[3,-1], pch=18, add = T, col=2, errbar.col=2,
cap = .05, lty=2)
errbar(x = at2, y = q_popt_y40[2,-1], yminus = q_popt_y40[1,-1], yplus = q_popt_y40[3,-1],pch = 17, add = T,
col=adjustcolor("blue", offset = c(0,.25,.25,.25)), errbar.col=adjustcolor("blue", offset = c(0,.25,.25,.25)),  cap = .05, lty=2)
lines(at1, q_p_y40[2,-1], type="b", col="green2", lwd=2, cex=1.5, pch=16)
lines(at4, q_t_y40[2,-1],   pch=15, type="b", col=1, lwd=2, cex=1.5)
lines(at3, q_pt_y40[2,-1],   pch=18, type="b", col=2, lwd=2, cex=1.5)
lines(at2, q_popt_y40[2,-1], pch=17, type="b", col=adjustcolor("blue", offset = c(0,.25,.25,.25)), lwd=2, cex=1.5)
axis(2, at=seq(0,1,.2), labels = rep("", 6))
dev.off()
```

```
# produce table with proportions of cases with found subgroups
table <- t(data.frame(sapply(d, function(k) round(mean(found_p5[delta==k]), 2))))
table <- rbind(table, sapply(d, function(k) round(mean(found_popt5[delta==k]), 2)))
table <- rbind(table, sapply(d, function(k) round(mean(found_pt5[delta==k]), 2)))
table <- rbind(table, sapply(d, function(k) round(mean(found_t5[delta==k]), 2)))
table <- rbind(table, sapply(d, function(k) round(mean(found_p5m[delta==k]), 2)))
table <- rbind(table, sapply(d, function(k) round(mean(found_popt5m[delta==k]), 2)))
table <- rbind(table, sapply(d, function(k) round(mean(found_pt5m[delta==k]), 2)))
table <- rbind(table, sapply(d, function(k) round(mean(found_t5m[delta==k]), 2)))
table <- rbind(table, sapply(d, function(k) round(mean(found_p20[delta==k]), 2)))
table <- rbind(table, sapply(d, function(k) round(mean(found_popt20[delta==k]), 2)))
table <- rbind(table, sapply(d, function(k) round(mean(found_pt20[delta==k]), 2)))
table <- rbind(table, sapply(d, function(k) round(mean(found_t20[delta==k]), 2)))
table <- rbind(table, sapply(d, function(k) round(mean(found_p40[delta==k]), 2)))
table <- rbind(table, sapply(d, function(k) round(mean(found_popt40[delta==k]), 2)))
table <- rbind(table, sapply(d, function(k) round(mean(found_pt40[delta==k]), 2)))
table <- rbind(table, sapply(d, function(k) round(mean(found_t40[delta==k]), 2)))
colnames(table) <- round(d,2)
table <- cbind(rep(c("PRIM", "PRIM opt.", "CART(pruned)", "CART(maxdepth)"), 4), table)
table <- cbind(c("5%", "", "", "", "5% (margin)","", "", "", "20%", "", "", "", "40%", "", "", ""), table)
row.names(table)<- NULL
table
```

Appendix G

```
# choose path for saving the images
# setwd("yourpath")

### install package PRIM (can be skipped if already done)
#install.packages("devtools")
#library(devtools)
#install_github("ao90/PRIM")

library(PRIM); library(rpart); library(rpart.plot); library(mlbench); library(party)
data(PimaIndiansDiabetes2)

# data overview
postscript("diabetes.eps",  width=7.5, height=7.5, horizontal = F, paper = "special")
plot(PimaIndiansDiabetes2[,-9], col=ifelse(PimaIndiansDiabetes2$diabetes=="pos", "grey20", "lightblue3"),
pch=ifelse(PimaIndiansDiabetes2$diabetes=="pos", 21, 3), bg="red", gap=0, oma=c(4,6,4,4), cex=.7)
legend("topleft", legend = c("neg", "pos"), col=c( "lightblue3", "grey20"),pt.bg="red" , xpd=NA, cex=.75, inset =
c(0.0175,.07), title="diabetes", pch=c(3,21), pt.cex = .7)
dev.off()

# apply peeling and plot the trajectories
peel <- PRIM_peel(formula = diabetes=="pos" ~ ., data=PimaIndiansDiabetes2) # singular peeling
peel1 <- PRIM_peel_bs(formula = diabetes=="pos" ~ ., data=PimaIndiansDiabetes2, seed = 123, B=10, peel_alpha =
seq(0.01, 0.5, 0.01)) # multiple peeling (takes a moment)
peel1_nd <- remove_dominated(peel1) # remove dominated boxes of multiple output

# Plot the two trajectories
postscript("diabetes_traj.eps", width=7.5, height=7.5, horizontal = F, paper = "special")
plot(peel1_nd$beta, peel1_nd$f, col=0, las=1, xlab=expression(beta), ylab="mean(y)")
grid(col="grey60")
points(peel1_nd$f~peel1_nd$beta, pch=21, bg=adjustcolor("red", offset=c(0,.2,.2,.2)), col="grey20", cex=2)
points(peel$f~peel$beta, pch=16, cex=2)
legend("topright", legend=c("singular traj.", "multiple traj."),col=c(1, "grey20"), pch=c(16,21), pt.bg=adjustcolor("red",
offset=c(0,.2,.2,.2)), bty = "n", pt.cex = 2)
dev.off()

# apply PRIM (with covering) to the data (can take about 10 minutes)
prim <- PRIM(formula = diabetes=="pos" ~ ., data=PimaIndiansDiabetes2, seed = 123, beta_min = 20/768, f_min=.8, B=10,
peel_alpha = seq(0.01, 0.5, 0.01))
prim$f
prim$beta
prim$box

# calculate mean of all observations not included in the first box
mean(prim$data_orig$`diabetes == "pos"`[prim$fixboxes[[1]]$subset==FALSE])
# number of observations in the boxes
prim$beta*nrow(PimaIndiansDiabetes2)


# apply CART with graphical illustration
set.seed(123)
tree <- rpart(diabetes=="pos" ~ ., data=PimaIndiansDiabetes2)

# pruning according to the 1-SE rule
min_x <- which.min(tree$cptable[,4]) # find the minimum crossvalidated error
border <- tree$cptable[min_x,4] + tree$cptable[min_x,5] # add 1 se to the minimum x_error
cp <- tree$cptable[tree$cptable[,4] < border,][1,1] # find the first cp value with xerror < border
tree_pruned <- prune(tree, cp = cp)

postscript("diabetes_tree.eps", width=7.5, height=5, horizontal = F, paper = "special")
rpart.plot(tree_pruned,extra=1, under=T,fallen.leaves=FALSE, type = 2, xlim=c(0,1), ylim=c(0,1), box.palette = "Reds")
dev.off()
```

Appendix F

```
### install package PRIM (can be skipped if already done)
#install.packages("devtools")
#library(devtools)
#install_github("ao90/PRIM")

library(PRIM)
library(survival)
library(party)

# to read the data set whitehall1 you have to download it online from "http://portal.uni-freiburg.de/imbi/Royston-Sauerbrei-book/index.html#datasets"
# setwd("yourpath") # choose the path in which the file whitehall1.csv is saved
dat <- read.csv("whitehall1.csv", header = T)

# apply multiple peeling and remove the dominated boxes
peel <- PRIM_peel_bs(Surv(pyar, chd)~ cigs + map + age + ht + wt + chol + as.factor(jobgrade), data=dat, seed = 123, B=5, beta_min = 0.01, peel_alpha=seq(.01,.31,.02))
#plot(peel)
peel_nd <- remove_dominated(peel)
#plot(peel_nd)

# proportions of box boundaries from all relevant boxes
sort(apply(peel_nd$box_metric, MARGIN = 2, function(x) sum(is.finite(x)))/nrow(peel_nd$box_metric))
sort(apply(peel_nd$box[13:16], MARGIN = 2, function(x) sum(x==F))/nrow(peel_nd$box[13:16]))
```
